# Supplementary material for: P-selectin axis plays a key role in microglia immunophenotype and glioblastoma progression
Source: Nat Commun. 2021 Mar 26;12:1912. doi: 10.1038/s41467-021-22186-0 (PMC7997963; doi:10.1038/s41467-021-22186-0)
Supplement: Supplementary file 1 — Supplementary Information [file 41467_2021_22186_MOESM1_ESM.pdf]

Supplementary Figure 1. Molecular and functional characterization of human and murine microglia.

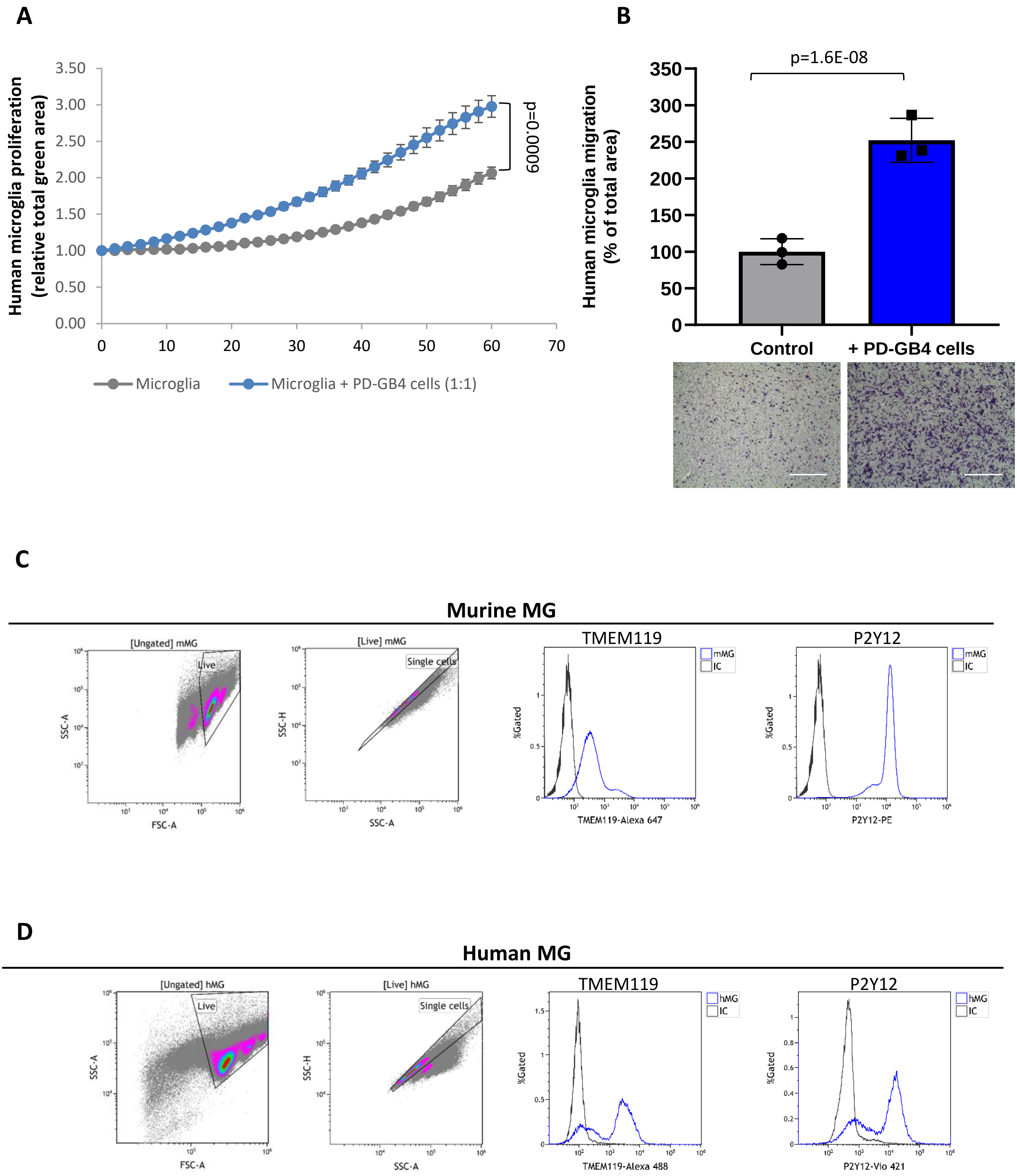

Supplementary Figure 1. Molecular and functional characterization of human and murine microglia. A-B. GB cells enhance the prolifartion and migration rate of human microglia cells A. Proliferation of GFP-labeled human microglia, showing enhanced proliferation of microglia cells when co-cultured with PD-GB4 GB cells (ratio of 1:1) compared to microglia monoculture. Images and analyses were obtained using the IncuCyte imaging system. Data represent mean ± s.d. of triplicate wells. The graph is a representative of three independent repeats. Statistical significance was determined using an unpaired, two-sided Student's *t*-test. **B.** Results and representative images of TransWell migration assay showing enhanced migration of human microglia cells towards PD-GB4 GB cells compared to naïve DMEM. Data represent mean ± s.d. of triplicate wells. The graph is a representative of three independent repeats. Statistical significance was determined using an unpaired, two-sided Student's *t*-test. Scale bars represent 400 μm**. C-D. Freshly-Isolated murine CD11b-positive cells and purchased human microglia are positive for microglia specific markers.**Flow cytometry analysis showing positive staining for TMEM119 and P2Y12 in isolated murine CD11b positive cells (C) and primary human microglia (D) .Source data are provided as a Source Data file.

Supplementary Figure 2. SELP has a role in GB-microglia interactions in GL261 and U251 GB models

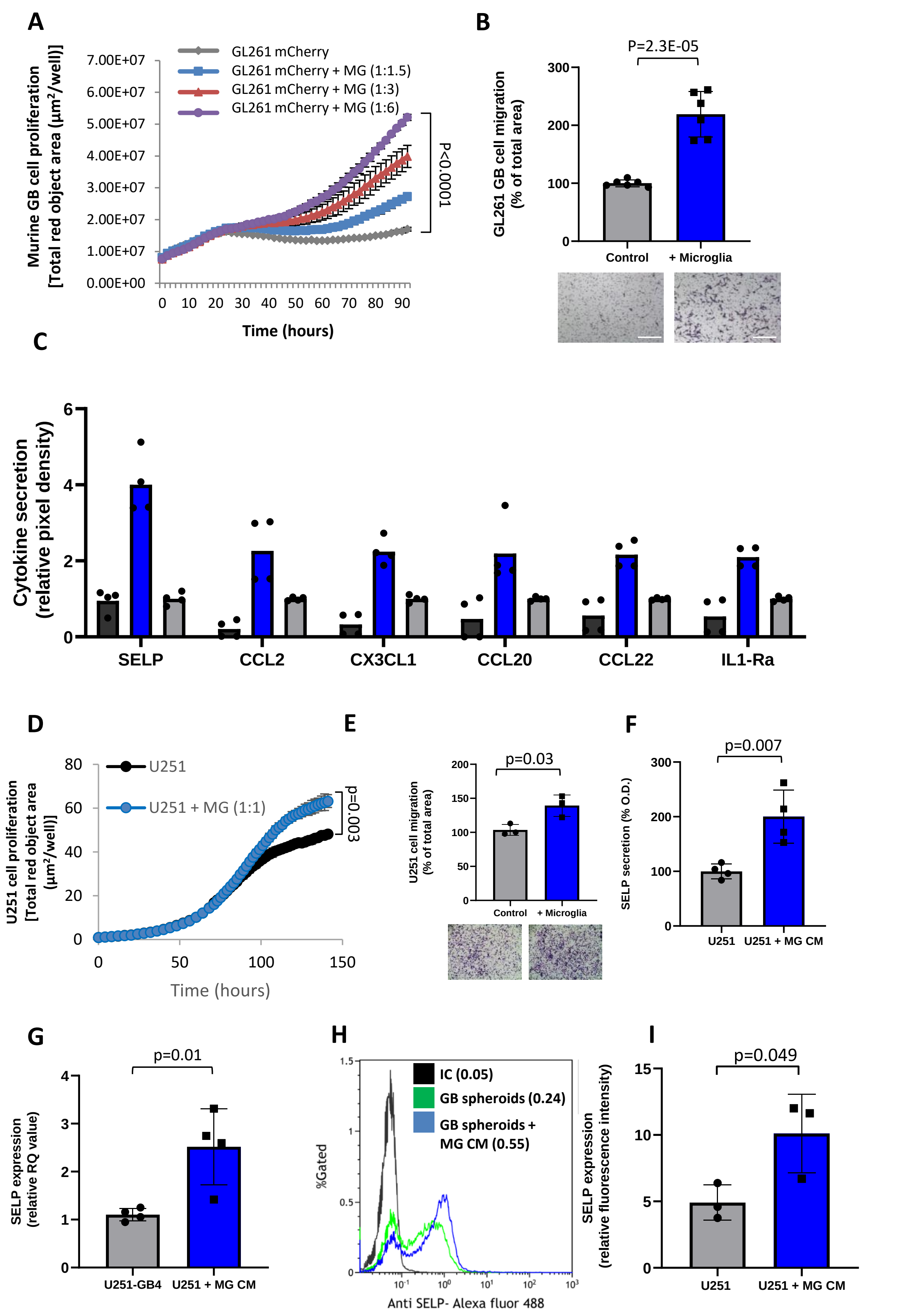

Supplementary Figure 2. SELP has a role in GB-microglia interactions in GL261 and U251 GB models. A. The Proliferation rate of mCherry-labeled GL261 GB cells rose as the concentration of microglia in the culture increased. Data represent mean ± s.d. of triplicate wells. The graph is a representative of three independent repeats. Statistical significance was determined using two ways ANOVA test with multiple comparisons adjustment. **B.** Transwell migration assay showing enhanced migration of murine GL261 GB cells towards primary murine microglia compared to naïve microglia medium. Data represent mean ± s.d. of six wells per group. The graph is a representative of three independent repeats. Statistical significance was determined using an unpaired, two-sided Student's *t*-test. Scale bars represent 400 µm**. C.** Cytokine profile showing over-secretion of SELP and other factors in a co-culture of murine GL261 GB cells and primary murine microglia compared to mono-cultures. Duplicates were measured for each cytokine. The graph shows the average of two independent studies including internal repeats. **D.** The Proliferation rate of iRFP-labeled human U251 GB cells was enhanced when co-cultured with human microglia compared to GB monoculture. Data represent mean ± s.d. of triplicate wells. The graph is a representative of three independent repeats. Statistical significance was determined using an unpaired, two-sided Student's *t*-test. **E.** Transwell migration assay showing enhanced migration of human U251 GB cells towards primary murine microglia compared to naïve microglia medium. Data represent mean ± s.d. of triplicate wells. The graph is a representative of three independent repeats. Statistical significance was determined using an unpaired, two-sided Student's *t*-test. Scale bars represent 400 µm. **F-G.** ELISA (F) and Real-time PCR (G) results showing over-secretion and mRNA expression of SELP by U251 GB cells when treated with microglia CM (MG CM) compared to naïve microglia medium. Data represent mean ± s.d. of four wells per group. The graphs are representative of three independent studies. Statistical significance was determined using an unpaired, two-sided Student's *t*-test. **H-I.** Representative image (H) and quantification (I) of flow cytometry analysis showing higher expression of SELP in U251 spheroids, treated with MG CM compared to naïve microglia medium. Data represent mean ± s.d. The graph shows the average of three independent repeats. Statistical significance was determined using an unpaired, two-sided Student's *t*-test. Source data are provided as a Source Data file.

Supplementary Figure 3. Immunostaining of GB mouse models, H&E of GB patient samples and GB spheroids FACS gating strategy

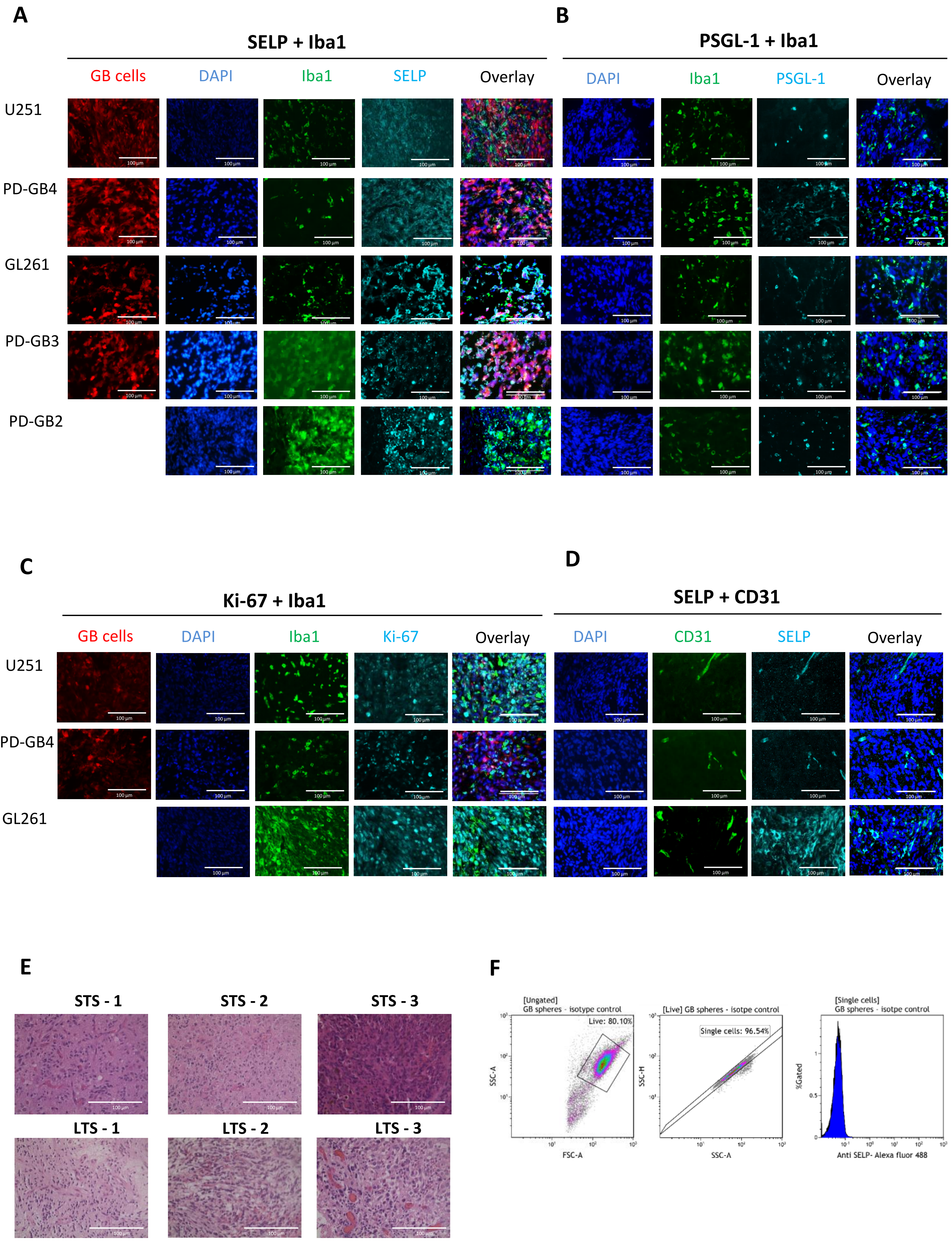

Supplementary Figure 3. Immunostaining of GB mouse models, H&E of GB patient samples and GB spheroids FACS gating strategy. A-B. Immuno-fluorescence staining presented as separate fluorescence channels, showing the co-staining for Iba1 and SELP/PSGL-1 in different GB mouse models showed in Figure 1 and in two additional PDX GB mouse models. N = 3 mice per staining. **C-D.** Immuno-fluorescence staining presented at separate fluorescence channels, showing co-staining for Iba1 + Ki-67 (C) and SELP + CD31 (D) in U251, PD-GB4 and GL261 GB mouse models. N = 3 mice per staining. **E.** Representative H&E staining of GB patients FFPE samples. N = 3 patient samples per group. **F.** FACS gating strategy used for the evaluation of SELP expression in GB spheroids shown in Figure 1G, 2B and Supplementary Figure 2H. All scale bars represent 100 µm**.**

Supplementary Figure 4. Over secreted cytokines in GB-microglia co-culture identified by human and murine cytokine arrays

A

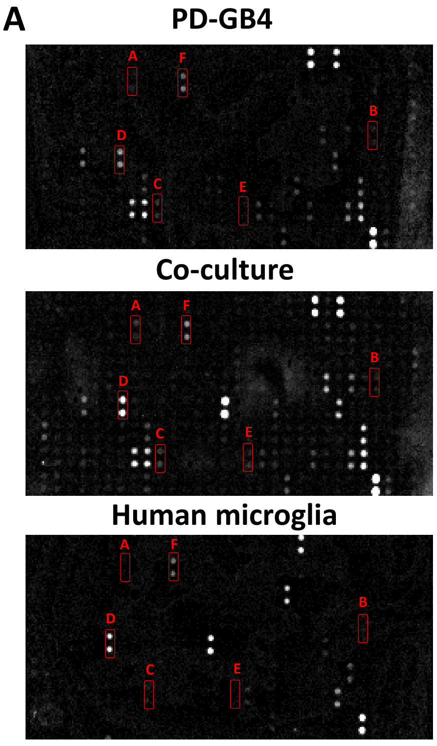

B

Reference	Cytokine	Id
A	IL-1ra	IL1RN
B	CX3CL1	CX3CL1
C	SELP	SELP
D	CCL20	CCL20
E	TGF-β	TGFB1
F	GLUT3	SLC2A3

C

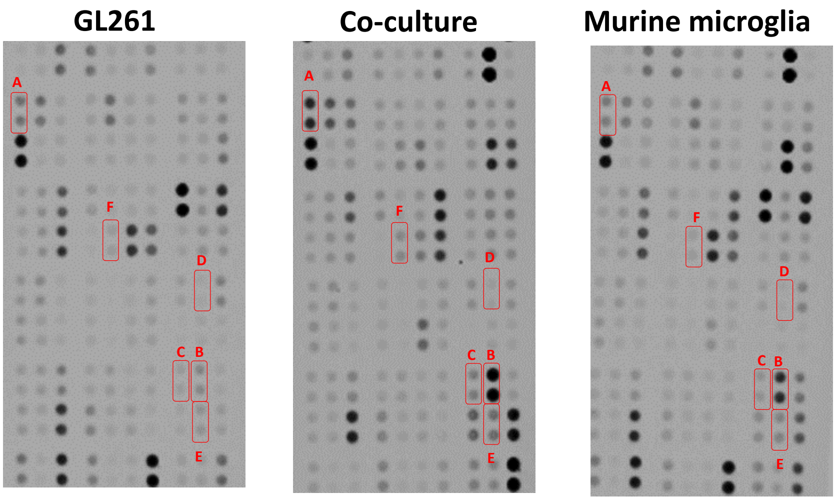

D

Reference	Cytokine	Id
A	SELP	Selp
B	CCL2	Ccl2
C	CX3CL1	CX3CL1
D	CCL20	Ccl20
E	CCL22	Ccl22
F	IL-1ra	Il1rn

Supplementary Figure 4. Over secreted cytokines in GB-microglia co-culture identified by human and murine cytokine arrays. A, C. Human (A) and murine (C) cytokine arrays membranes showing in red the over secreted factor in a co-culture of GB cells and microglia compared to monocultures. **B, D.** Gene IDs of over secreted factors detected by human (B) and murine (D) cytokine arrays.

Supplementary Figure 5. Pharmacological or molecular inhibition of SELP leads to a decrease in sprouting and invasion capabilities of a number of GB-spheroids.

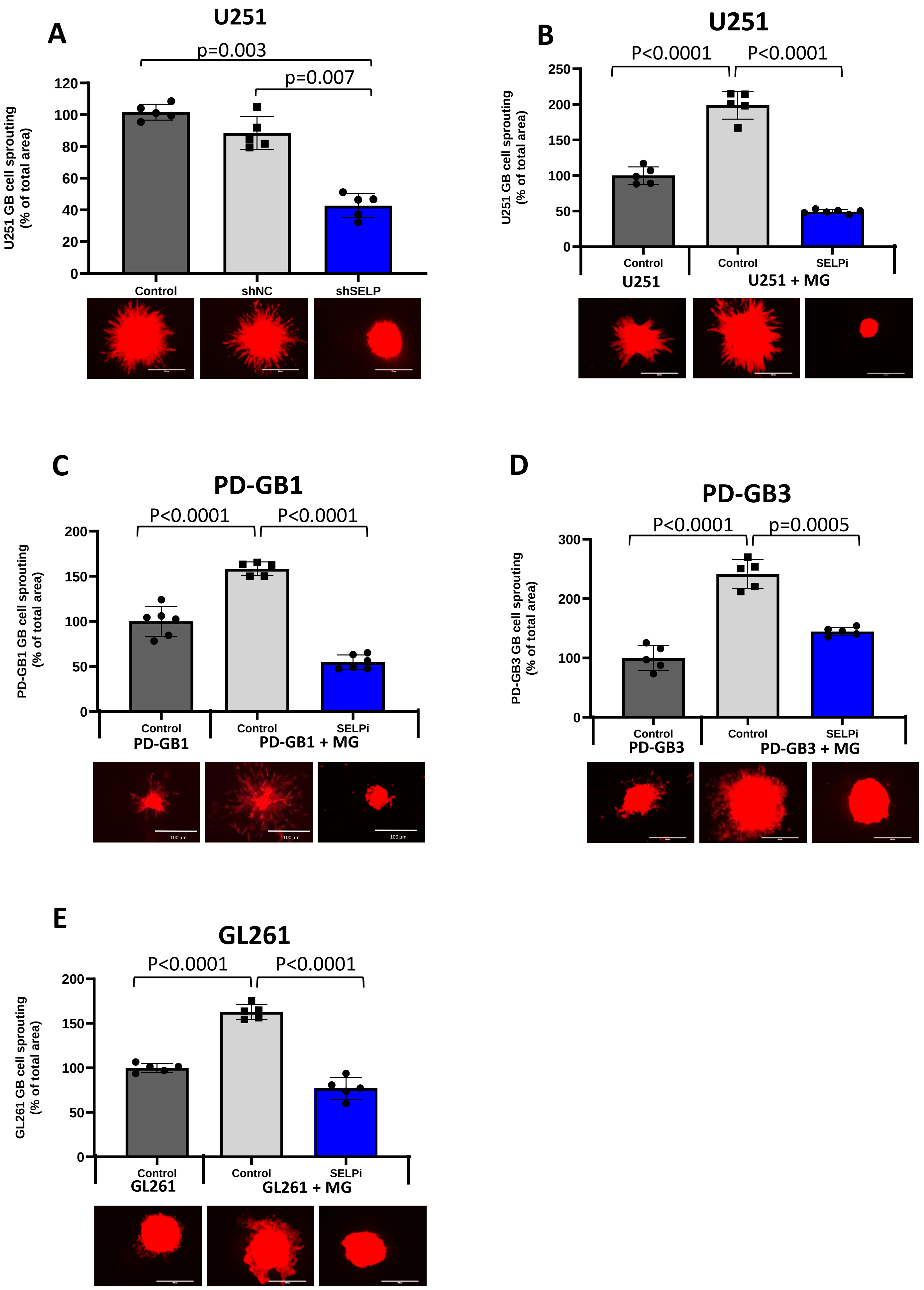

Supplementary Figure 5. Pharmacological or molecular inhibition of SELP leads to a decrease in sprouting and invasion capabilities of a number of GB-spheroids. A. Spheroids of iRFP-labeled U251 cells, co-cultured with unlabeled human microglia, showing reduced invasion of shSELP U251 cells compared to control WT or shNC U251 cells. mean ± s.d. of five spheroids per group. The graphs is a representative three independent repeats. Statistical significance was determined using one way ANOVA test with multiple comparisons adjustment. Scale bars represent 100 µm. **B-E.** Spheroids of mCherry-labeled U251 (B), PD-GB1 (C), PD-GB3 (D) and GL261 (E) cells, alone and co-cultured with unlabeled microglia, untreated or treated with 0.5 µM SELPi. Cell sprouting was inhibited by SELPi treatment. Sprouting was monitored for 72 h. Data represent mean ± s.d. of five spheroids per group. The graphs of panels B and C are representative of 3 independent repeats, the graphs of panels D and E are representative of 2 independent repeats. Statistical significance was determined using one way ANOVA test with multiple comparisons adjustment. Scale bars represent 100 µm. Source data are provided as a Source Data file.

Supplementary Figure 6. Working concentrations of SELPi did not induce apoptosis in PD-GB4 and U251 GB cells

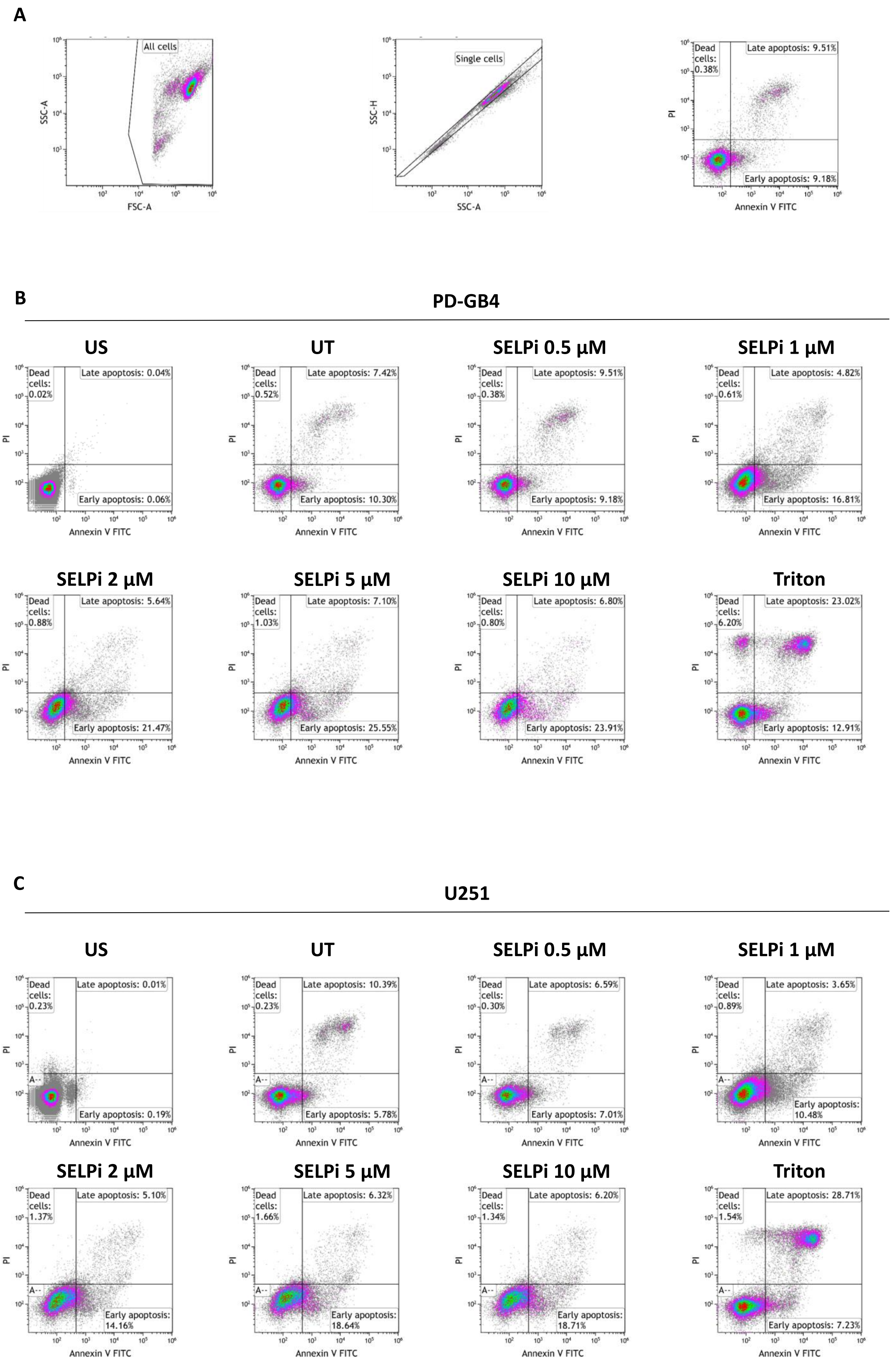

Supplementary Figure 6. Working concentrations of SELPi did not induce apoptosis in PD-GB4 and U251 GB cells. A. Example for gating strategy for apoptosis-necrosis evaluation by flow cytometry. **B-C.** Annexin-PI apoptosis-necrosis staining was performed using PD-GB4 (A) and U251 (B) cells. Mild induction of apoptosis was observed only when GB cells were treated with high concentrations of SELPi at 5 and 10 µM but not at 0.5 and 1 µM.

Supplementary Figure 7. SELP mediates GB – microglia interactions in different murine GB subtypes

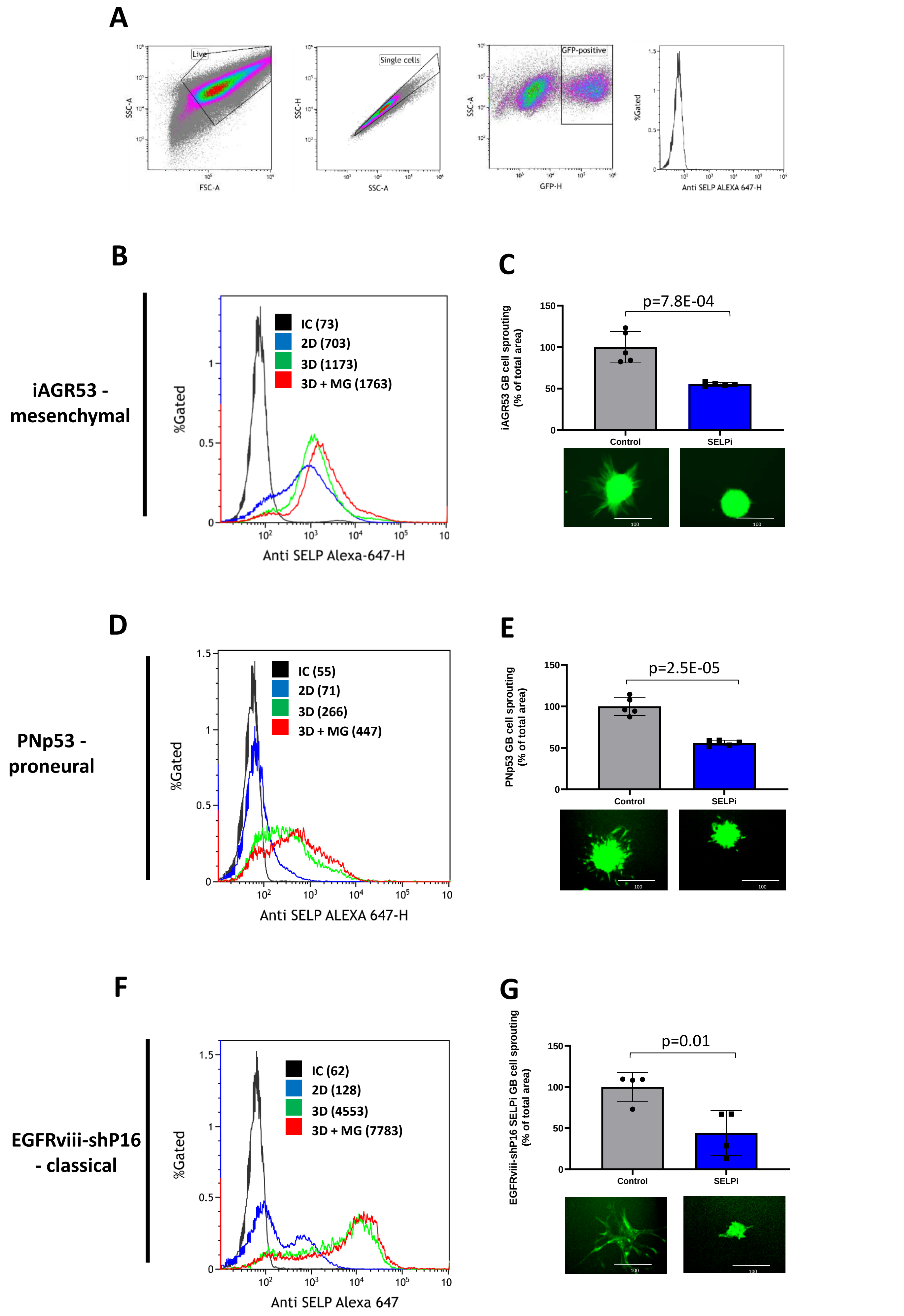

Supplementary Figure 7. SELP mediates GB – microglia interactions in different murine GB subtypes. A. Gating strategy for evaluation of SELP expression by GFP-labeled murine GB spheroids co-cultured with unlabeled murine microglia. **B,D,F.** Flow cytometry analysis showing increased expression of SELP by mesenchymal (A), proneural (C) and classical (E) murine GB cells when co-cultured with murine microglia. **C,E,G.** GB-microglia 3D spheroids showing inhibition of GB cell-sprouting when treated with SELPi using mesenchymal (B), proneural (D) and classical (F) murine GB subtypes. Data represent mean ± s.d. of five spheroids per group. The graph of panel C is a representative of 3 independent repeats, the graphs of panels E and G are representative of 2 independent repeats. Scale bars represent 100 µm. Statistical significance was determined using an unpaired, two-sided Student's *t*-test. Scale bars represent 100 µm. Source data are provided as a Source Data file.

Supplementary Figure 8. SELP mediates the expression of immune-related markers and induces the activation of the NF-k pathway in human microglia.

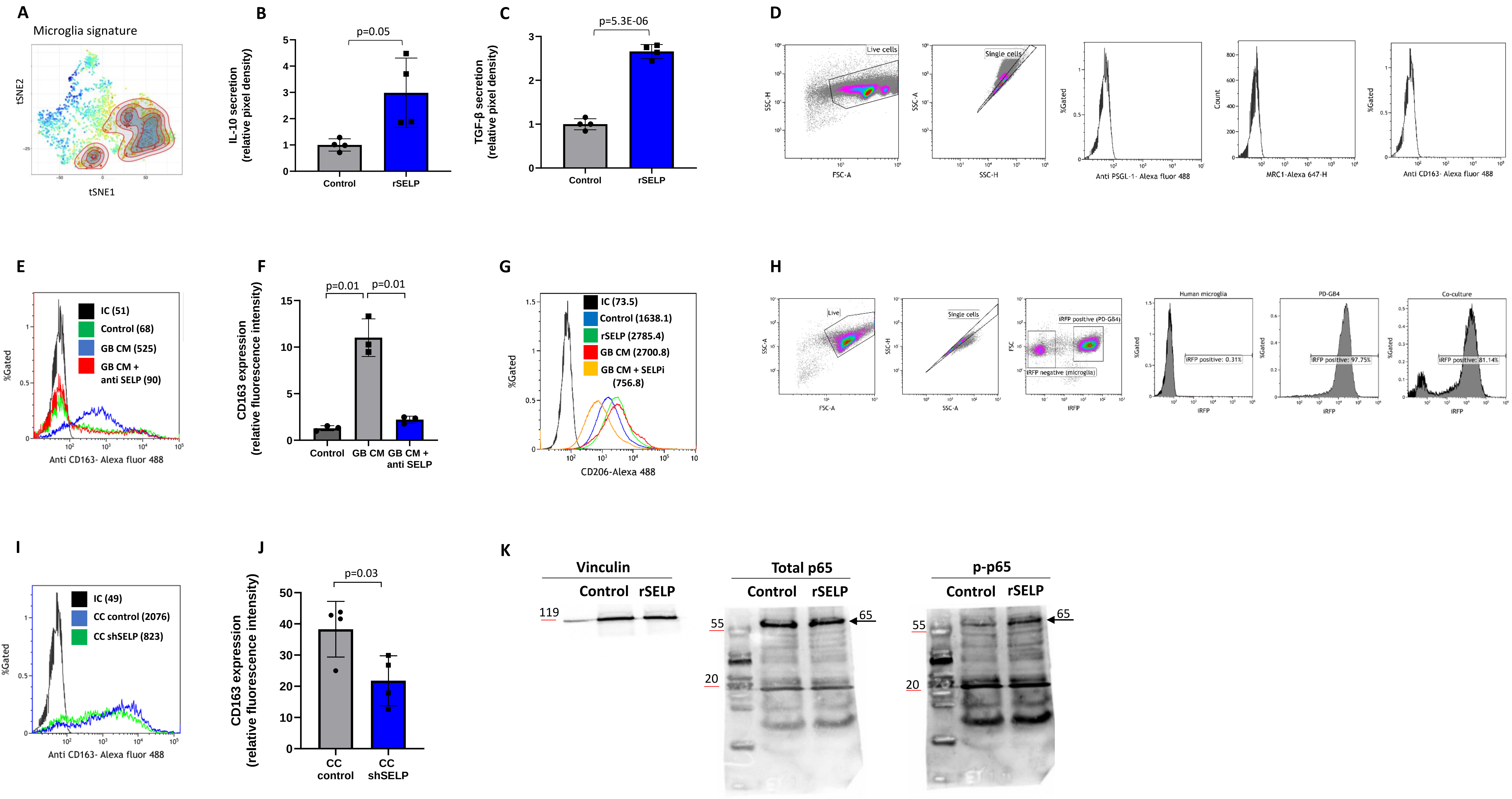

Supplementary figure 8. SELP mediates the expression of immune-related markers and induces the activation of the NF-k pathway in human microglia. A. Signature projection of microglia [96]. **B-C.** Secretion of IL-10 (B) and TGF-β (C) by human microglia was higher when treated with rSELP compared to untreated cells. Medium was analyzed by cytokine array. Data represent mean ± s.d. The graph shows the average of two independent repeats including internal repeats. Statistical significance was determined using an unpaired, two-sided Student's *t*-test. **D.** Example for Gating strategy for evaluation of PSGL-1 expression by human microglia shown in Figure 3C-D, and for CD163 and CD206 expression by human microglia when grown in 2D culture. **E-F.** Representative image (E) and quantification (F) of flow cytometry analysis of CD163 expression by human microglia. Microglia were grown for 48 h in 2D in a petri dish containing control medium, PD-GB4 CM, or PD-GB4 CM supplemented with SELP neutralizing antibody. The enhanced expression of CD163 in the presence of PD-GB4 CM was reduced when microglia were treated with SELP neutralizing antibody. Data represent the mean ± s.d. The graph shows the average of three independent experiments. Statistical significance was determined using one-way ANOVA test with multiple comparisons adjustment. **G.** Flow cytometry analysis showing high expression of CD206 by human microglia when exposed to rSELP and GB CM, and lower expression when SELPi was added to GB CM. **H.** Flow cytometry showing the gating strategy and the distinction between iRFP-labeled PD-GB4 and unlabeled human microglia isolated from 3D spheroids. **I-J.** Representative image (I) and quantification (J) of flow cytometry analysis of the expression of CD163 by human microglia revealed a lower expression of CD163 by microglia isolated from 3D spheroids containing shSELP PD-GB4 cells than spheroids containing control WT PD-GB cells. The spheroids were cultured for 48 h. Data represent mean ± s.d. The graph shows the average of four independent experiments. Statistical significance was determined using an unpaired, two-sided Student's *t*-test. **K.** Representative Western Blot showing elevated levels of phosphorylated p65 in human microglia following treatment for 3 h with rSELP compared to untreated cells. Source data are provided as a Source Data file.

Supplementary Figure 9. SELP mediates murine microglia immunophenotype

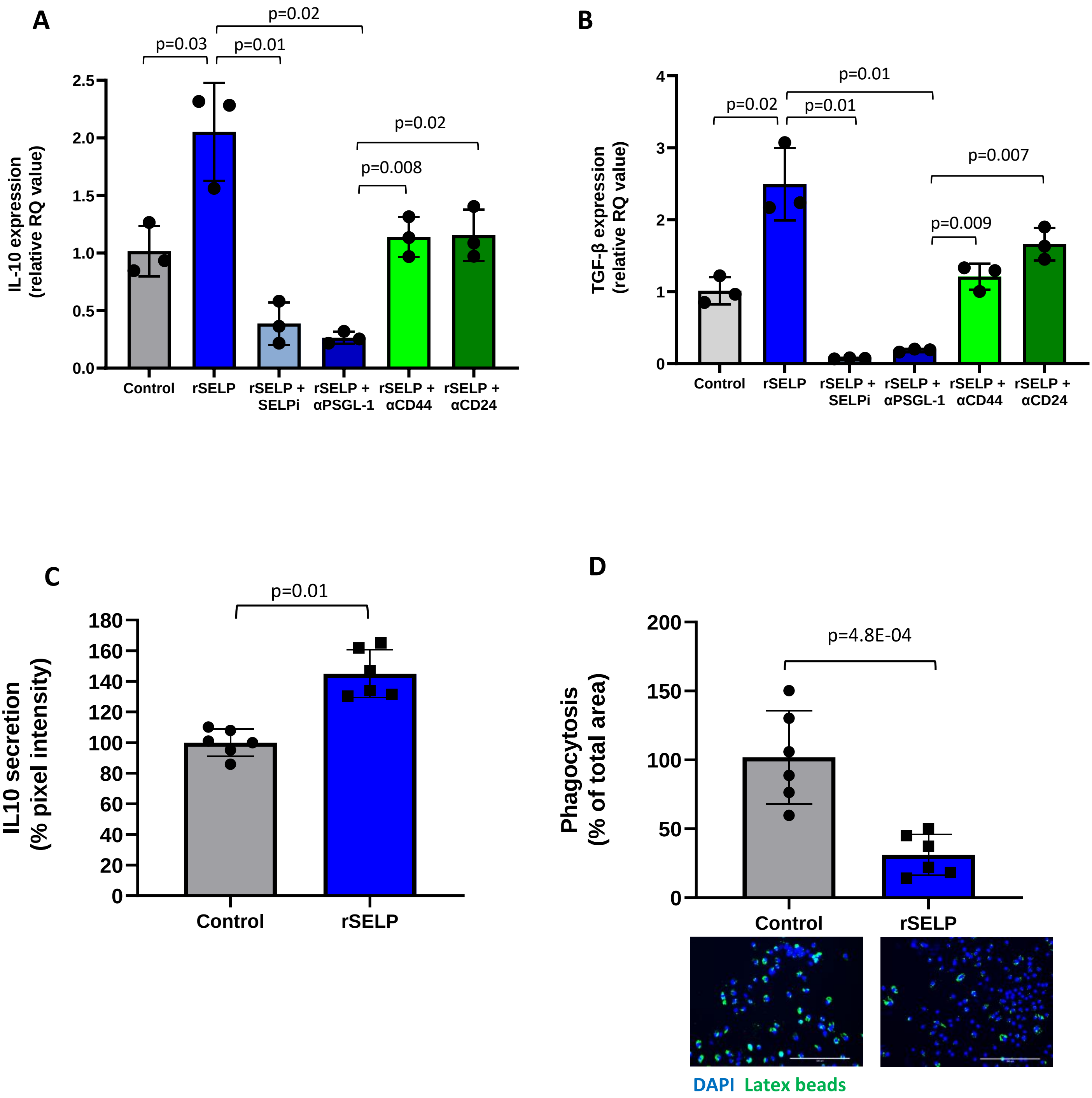

Supplementary Figure 9. SELP mediates murine microglia immunophenotype. A-B. Real-time PCR showing induction of IL-10 and TGF-β expression by murine microglia when treated with rSELP. IL-10 and TGF-β expression was reduced when SELPi or anti-PSGL-1 neutralizing antibody were added but only partially reduced when anti-CD44 or anti-CD24 neutralizing antibodies were added. Data represent mean ± s.d. of triplicates. Each graph is a representative of three independent repeats. Statistical significance was determined using one way ANOVA test with multiple comparisons adjustment. **C.** Secretion of IL-10 by murine microglia was higher when treated with rSELP compared to untreated cells. Medium was analyzed by cytokine array. Data represent mean ± s.d. Duplicates were measured for each biological repeat. The graph shows the average of three independent repeats including internal repeats. Statistical significance was determined using an unpaired, two-sided Student's *t*-test. **D.** Phagocytosis assay performed on murine microglia, showing reduced phagocytic abilities when treated with rSELP compared to untreated cells. Data represent mean ± s.d. of six wells per group. The graph is a representative of three independent studies. Statistical significance was determined using an unpaired, two-sided Student's *t*-test. Scale bars represent 200 µm. Source data are provided as a Source Data file.

Supplementary Figure 10. SELP mediates bone marrow – derived macrophages (BMDM) immunophenotype

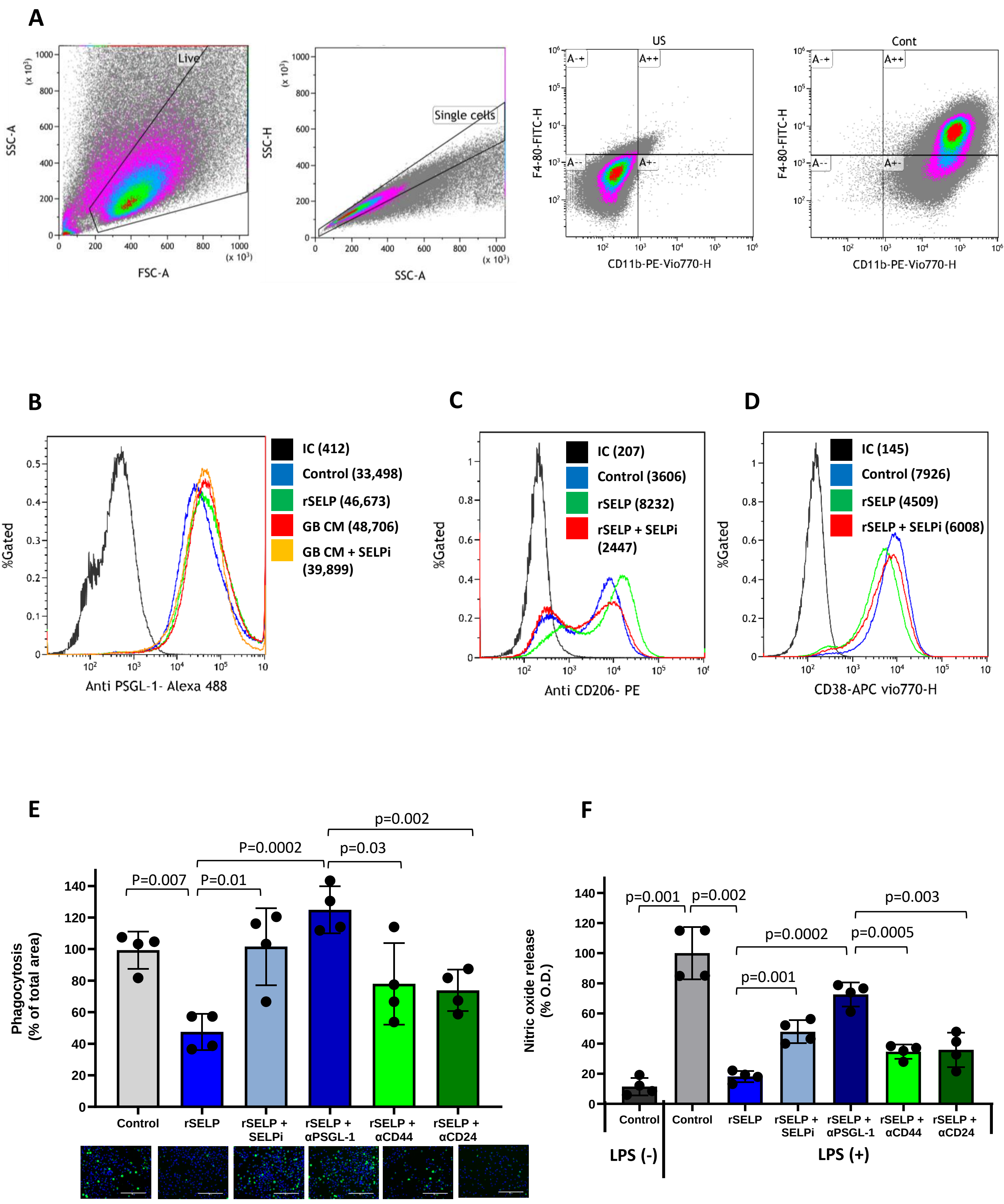

Supplementary Figure 10. SELP mediates BMDM immunophenotype. A. Flow cytometry analysis showing gating strategy for freshly isolated BMDM which are positive for the macrophages markers CD11b and F4/80. **B.** PSGL-1 expression by BMDM was increased following treatment with rSELP and GB CM, and was reduced when SELPi was added to GB CM. **C-D.** BMDM expressed higher levels of the anti-inflammatory marker CD206 (C) and lower levels of the pro-inflammatory marker CD38 (D) when treated with rSELP compared to untreated cells. CD206 expression was reduced while CD38 expression was elevated when SELPi was added to rSELP treatment. **E.** Phagocytic activity of BMDM was reduced following treatment with rSELP and GB CM. This activity was restored when SELPi or anti-PSGL-1 neutralizing antibody were added and only partially restored when anti-CD44 or anti-CD24 neutralizing antibodies were added. Data represent mean ± s.d. of four wells per group. The graph is a representative of three independent repeats. Scale bars represent 200 µm. Statistical significance was determined using one way ANOVA test with multiple comparisons adjustment. **F.** NO release by LPS stimulated BMDM was reduced when treated with rSELP, and was restored when SELPi or anti-PSGL-1 neutralizing antibody were added but not when anti-CD44 or anti-CD24 neutralizing antibodies were added. NO levels in the medium were analyzed by a total NO detection kit. Data represent mean ± s.d. of four wells per group. The graph is a representative of three independent experiments. Statistical significance was determined using one-way ANOVA test with multiple comparisons adjustment. Source data are provided as a Source Data file.

Supplementary Figure 11. PSGL-1 expression is positively correlated with the expression of microglia signature and CD4, and SELP is expressed by cancer cells while PSGL-1 IS expressed by microglia/macrophages and infiltrating T cells in GB patients

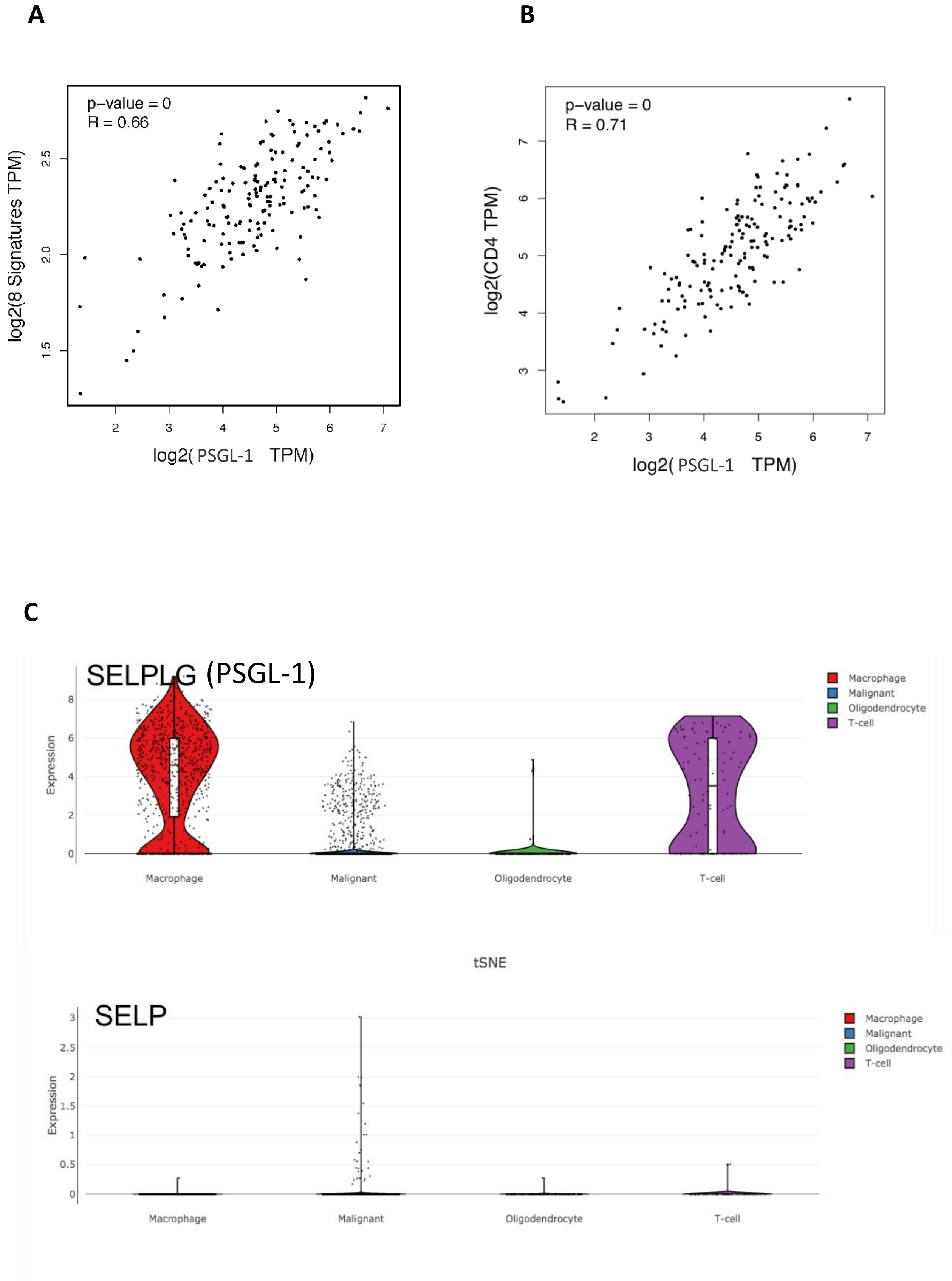

Supplementary Figure 11. PSGL-1 expression is positively correlated with the expression of microglia signature and CD4, and SELP is expressed by cancer cells while PSGL-1 IS expressed by microglia/macrophages and infiltrating T cells in GB patients. A-B. Analysis of TCGA and GTEx data, showing positive correlation between the expression of PSGL-1 (SELPLG) and the expression microglia gene signature - Tmem119, Cx3cr1, P2ry12, Arg1, Olfml3, Gpr34, Ccl4 and Ccl3 (A) and CD4 (B) in 163 GB patients. **C.** Analysis of single cell RNA-seq published data of 28 adult and pediatric GB samples [57], showing the expression of SELP and PSGL-1 in macrophages, tumor cells, oligodendrocytes, and T cells. Center of the box plots shows median values, boxes extent from 25% to the 75% percentile, whiskers show minimum and maximum values.

Supplementary Figure 12. SELP knockdown resulted in increased levels cytotoxic T cells and reduced levels of T regs in GL261 tumors

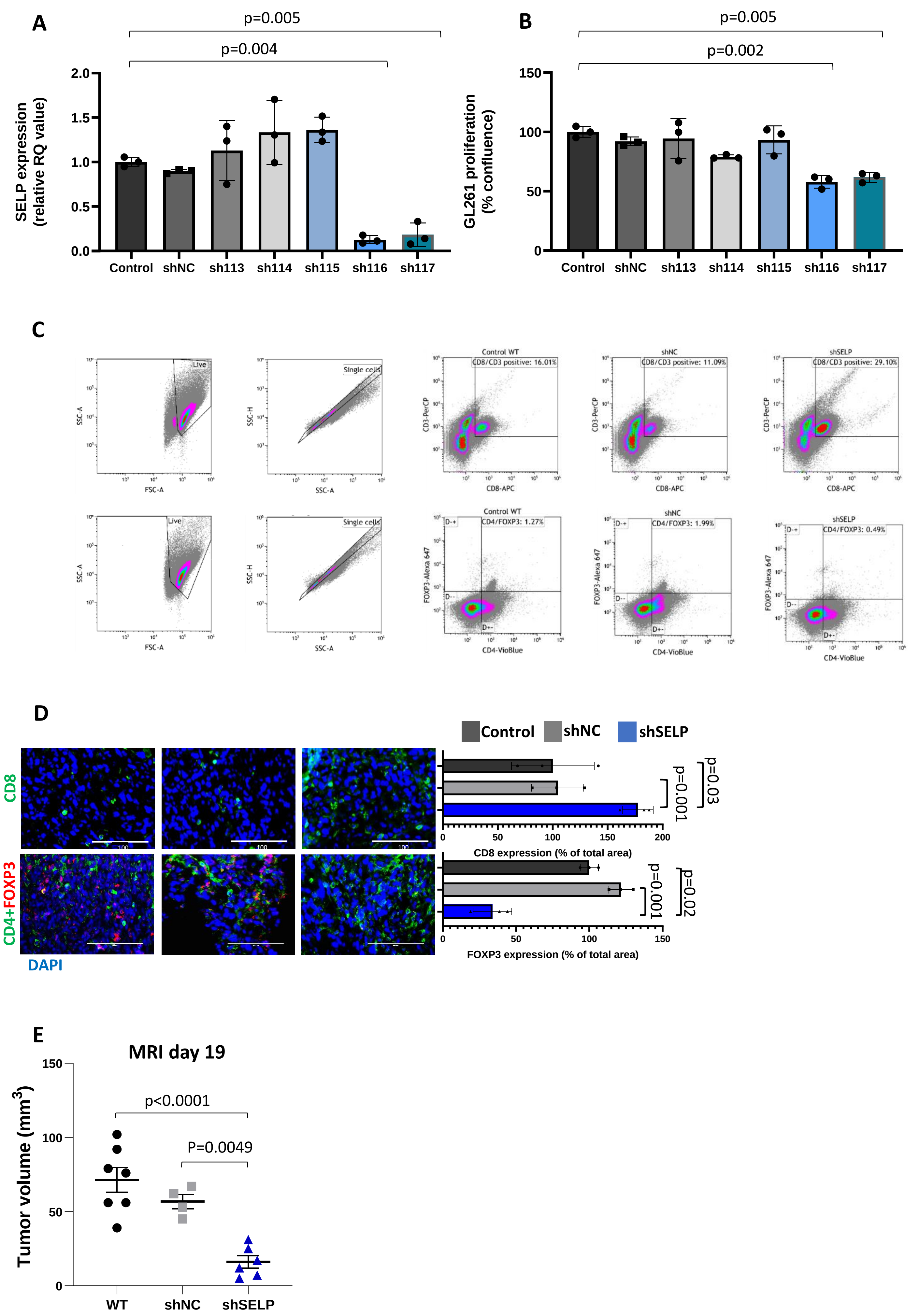

Supplementary Figure 12. SELP knockdown resulted in increased levels cytotoxic T cells and reduced levels of T regs in GL261 tumors. A. SELP knockdown in GL261 cells using different SELP shRNA plasmid. Data represent mean ± s.d. The graph is a representative of three independent experiments. Statistical significance was determined using one-way ANOVA test with multiple comparisons adjustment. **B.** GL261 proliferation following retro-viral infection of different SELP shRNA plasmids. Data represent mean ± s.d. The graph is a representative of three independent experiments. Statistical significance was determined using one way ANOVA test with multiple comparisons adjustment. **C.** Flow cytometry analysis showing high levels of CD3+CD8+ cells and low levels of CD4+FOXP3+ cells in shSELP GL261 tumors compared to control and shNC tumors. **D.** Immunostaining showing high levels of CD8 staining and low levels of FOXP3 staining in shSELP tumors compared to control and shNC tumors. Each dot represents the average of three images per mouse. Data represent mean ± s.d. N=3 mice per group. Statistical significance was determined using one-way ANOVA test with multiple comparisons adjustment. Scale bars represent 100 µm. **E.** Tumor volume detected by MRI (T1 weighted following Gd-DTPA administration), on day 19 post tumor inoculation, showing delayed tumor growth for shSELP group. Data represent mean ± s.e.m. N= 7 control, 4 shNC and 7 shSELP. Statistical significance was determined using one-way ANOVA test with multiple comparisons adjustment. Source data are provided as a Source Data file.

Supplementary Figure 13. Cell type specific gene expression and population distribution of different cell types between shNC and shSELP tumors, identified in whole-tumor single-cell RNA-seq

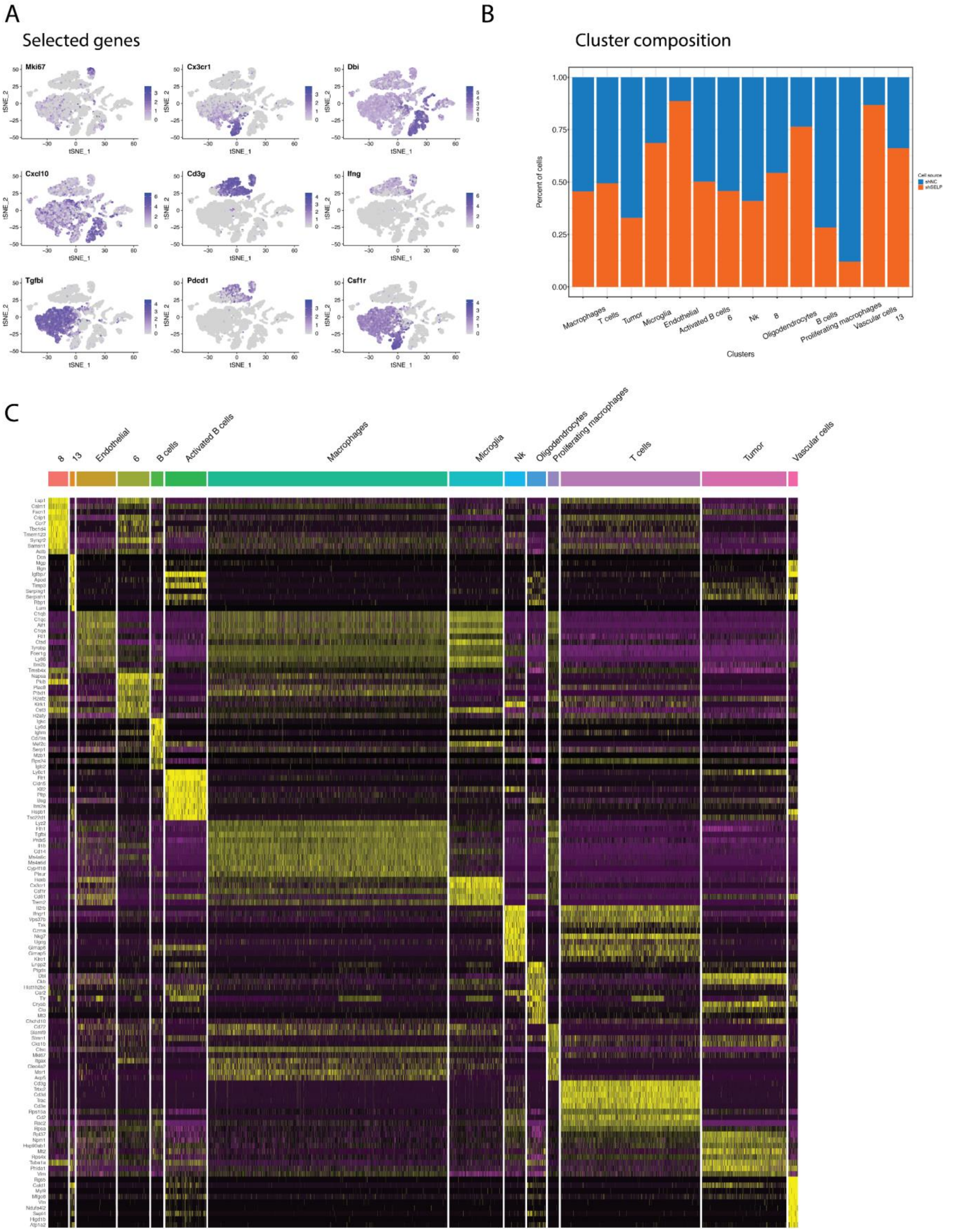

Supplementary Figure 13. Cell type specific gene expression and population distribution of different cell types between shNC and shSELP tumors, identified in whole-tumor single-cell RNA-seq. A. tSNE plots showing the expression of cell specific genes within the different clusters identified in GL261 tumors. **B.** Cell type clusters distribution between shNC and shSELP GL261 tumors. **C.** Heat map showing the ten most up-regulated differentially expressed genes in each cluster represent the different cell types identified in the tumors.

Supplementary Figure 14. Identified Tumor cell clusters found in whole-tumor single-cell RNA-seq and their distribution between shNC and shSELP GL261 tumors

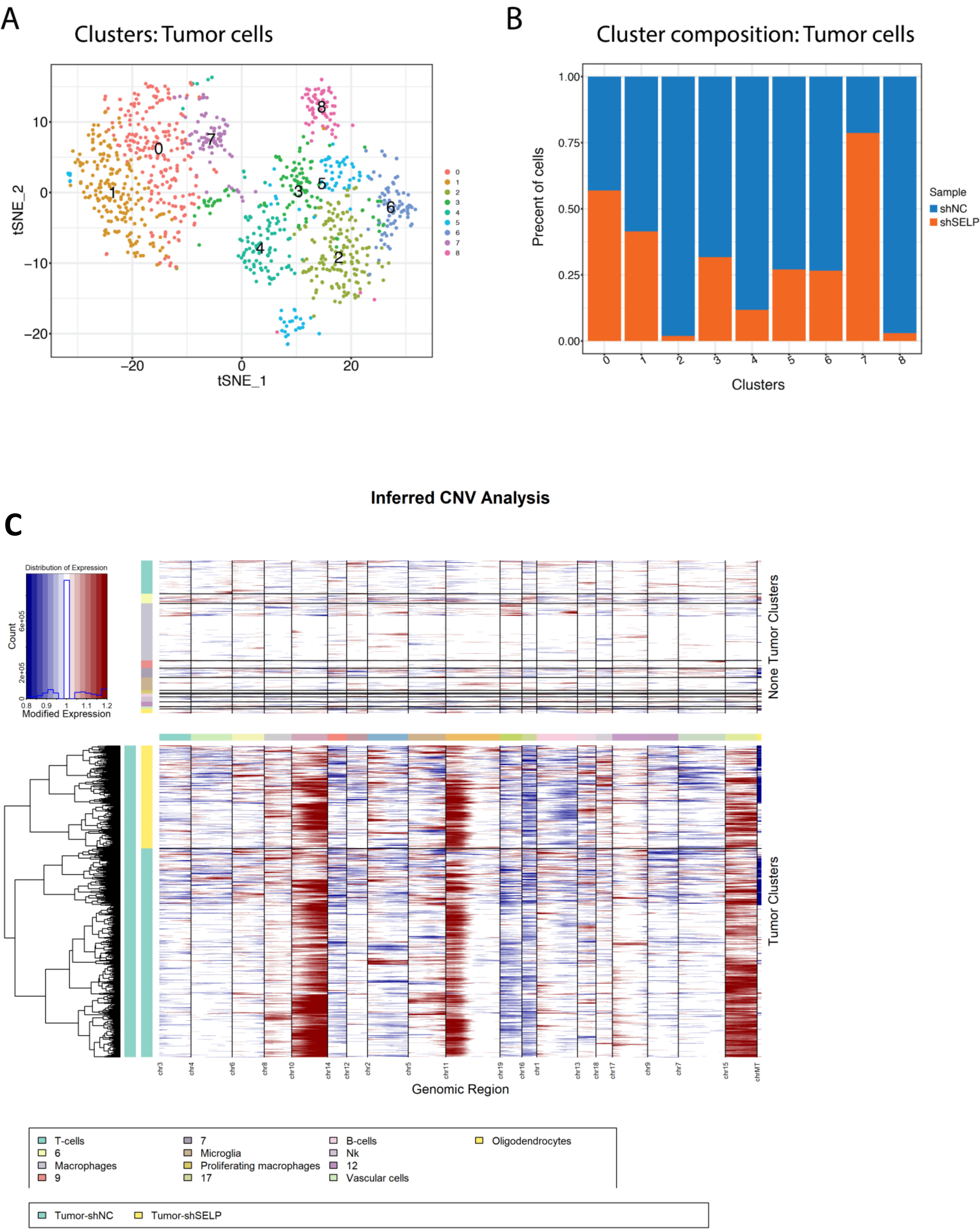

Supplementary Figure 14. Identified tumor cell clusters found in whole-tumor single-cell RNA-seq and their distribution between shNC and shSELP GL261 tumors. A. tNES plot showing the different tumor cell clusters identified within the tumor cell population in GL261 tumors. **B.** distribution of cancer cell clusters between shNC and shSELP GL261 tumors. **C.** Inferred CNV analysis. The analysis identified large multiploidy genomic region in the clusters annotated as tumor cells in both shSELP and shNC populations, while the rest of the clusters exhibited no such features.

Supplementary Figure 15. Microglia/macrophages populations and signatures identified in whole-tumor single-cell RNA-seq

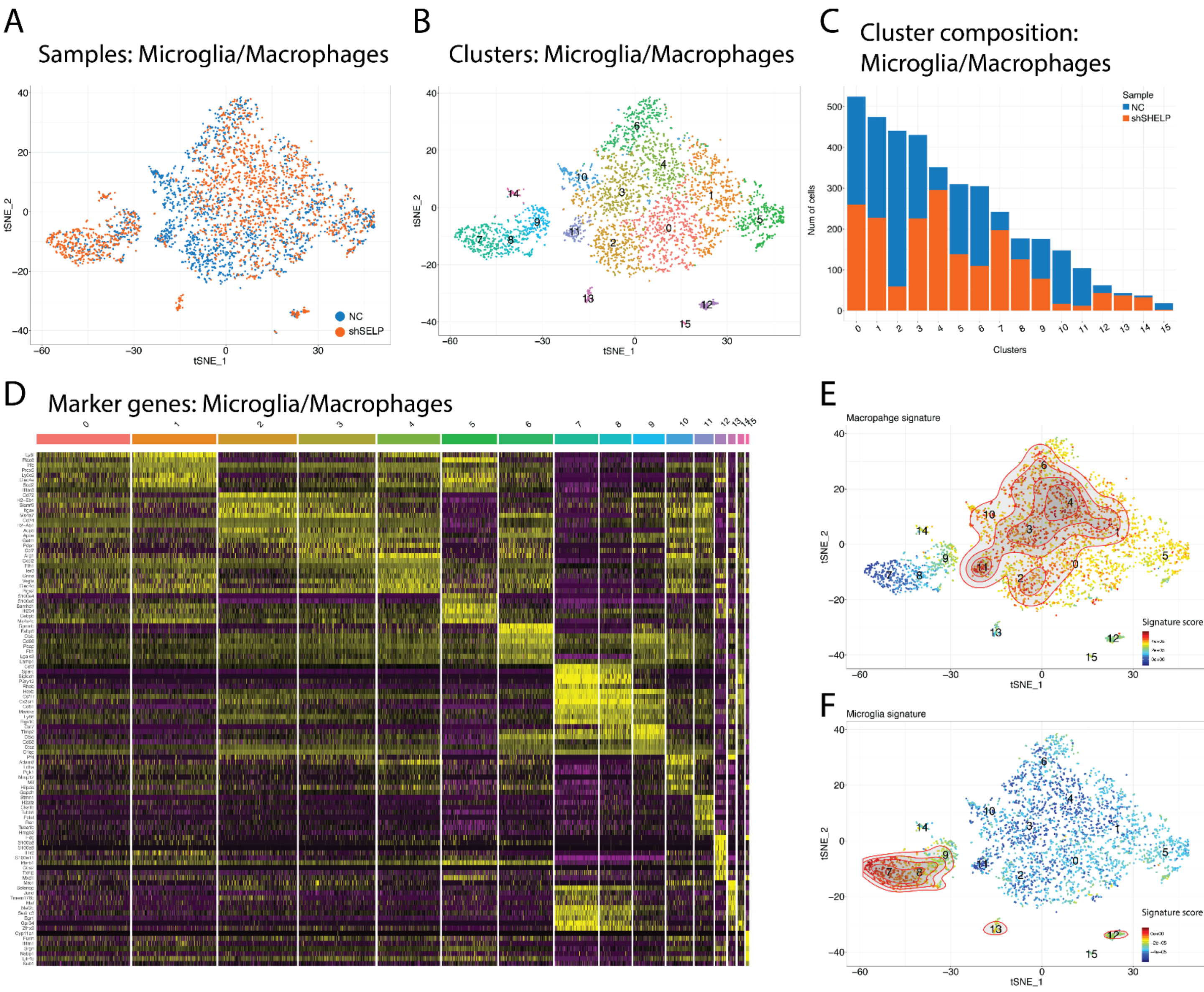

Supplementary Figure 15. Microglia/macrophages populations and signatures identified in whole-tumor single-cell RNA-seq. A-C. Microglia/macrophages Clusters identification and distribution identified in whole-tumor single-cell RNA-seq of shNC and shSELP GL261 tumors. **D.** Heat map showing the most differentially expressed genes in each microglia/macrophages cluster. **E-F.** Macrophages (E) and microglia (F) specific signatures expression.

Supplementary Figure 16: microglia/macrophages signatures and genes expression identified in Single-Cell RNA-seq of CD11b positive cells

A

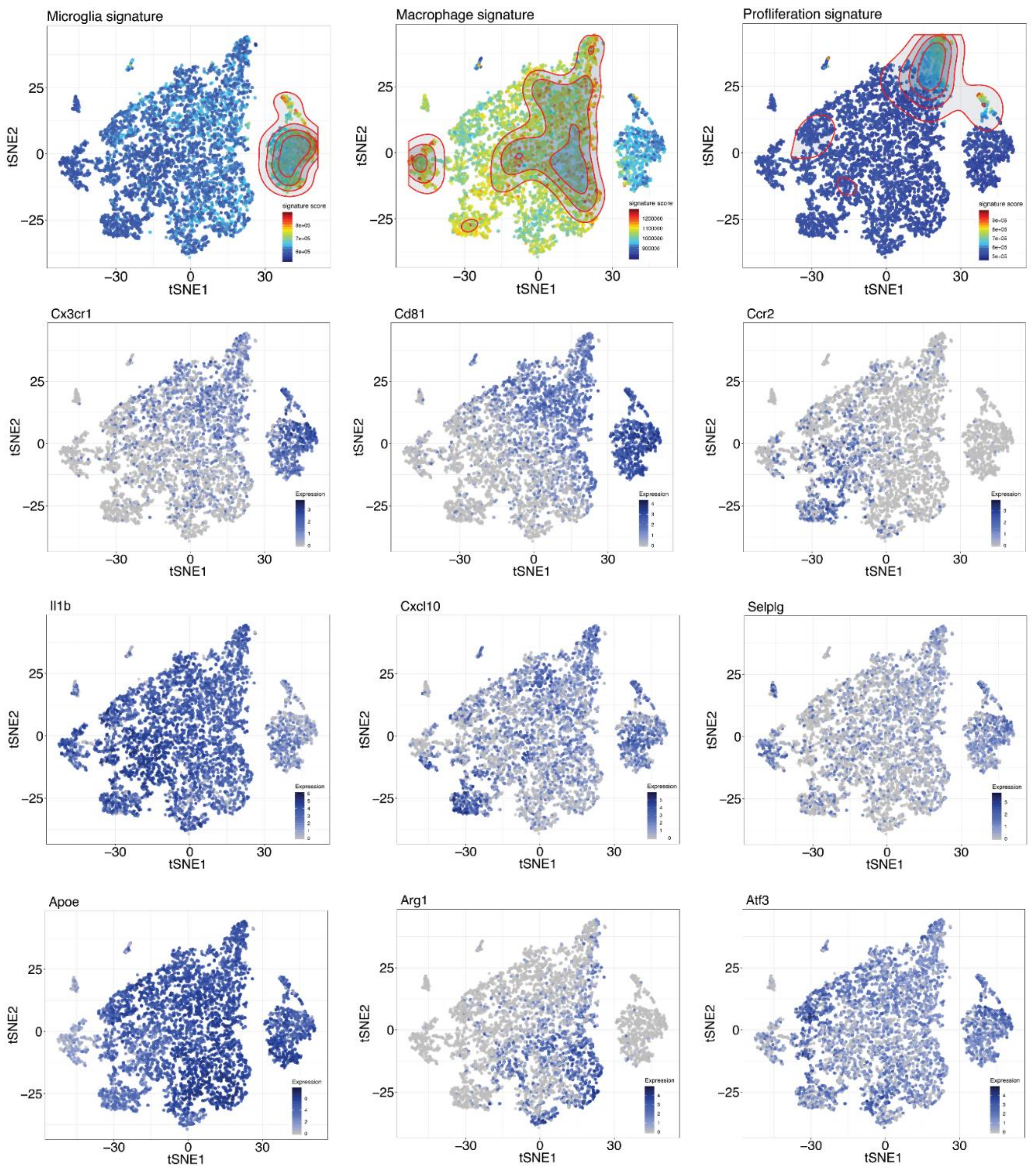

B

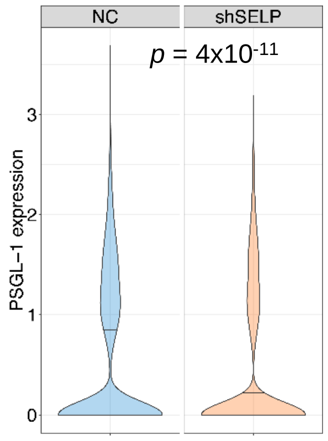

Supplementary Figure 16. Supplementary Figure 16: microglia/macrophages signatures and genes expression identified in Single-Cell RNA-seq of CD11b positive cells A. tSNE plot of single-cell RNA profiles of microglia/macrophage cells (CD11b+) from GL261 GB-bearing mice showing projection of microglia/macrophages [133] and proliferative cell [134] signatures, heat maps representing specific genes expressed by different microglia/macrophage sub-populations. **B.** Microglia isolated from shSELP GB tumors expressed lower levels of PSGL-1 compared to microglia isolated from control-WT tumors analyzed by single-cell RNA-seq. (*p* value = $4x10^{-11}$, unpaired, two-sided Student's *t*-test).

Supplementary Figure 17. T cell specific clusters, distribution, and differentially expressed genes in shNC and shSELP tumors

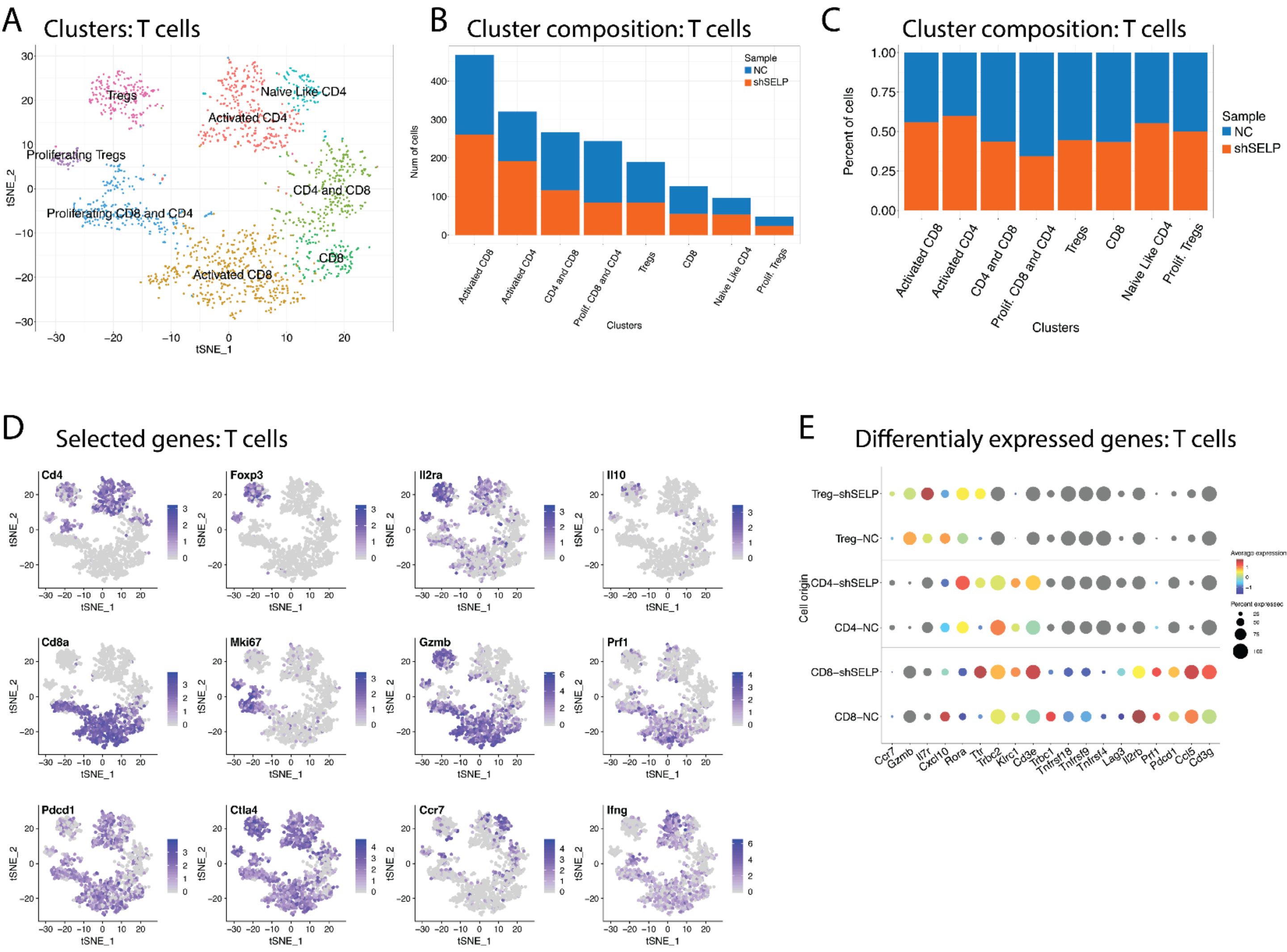

Supplementary Figure 17. T cell specific clusters, distribution, and differentially expressed genes in shNC and shSELP tumors. A-C. Different T cell clusters and their distribution in shNC and shSELP tumors, identified in whole-tumor single-cell RNA-seq. **D.** specific genes expression in the different identified T cell clusters. **E.** Differentially-expressed genes found in T cell clusters showing higher levels of T cell activation in shSELP tumors.

Supplementary Figure 18. SELPi treatment resulted in increased levels cytotoxic T cells and reduced levels of T regs in GL261 tumors

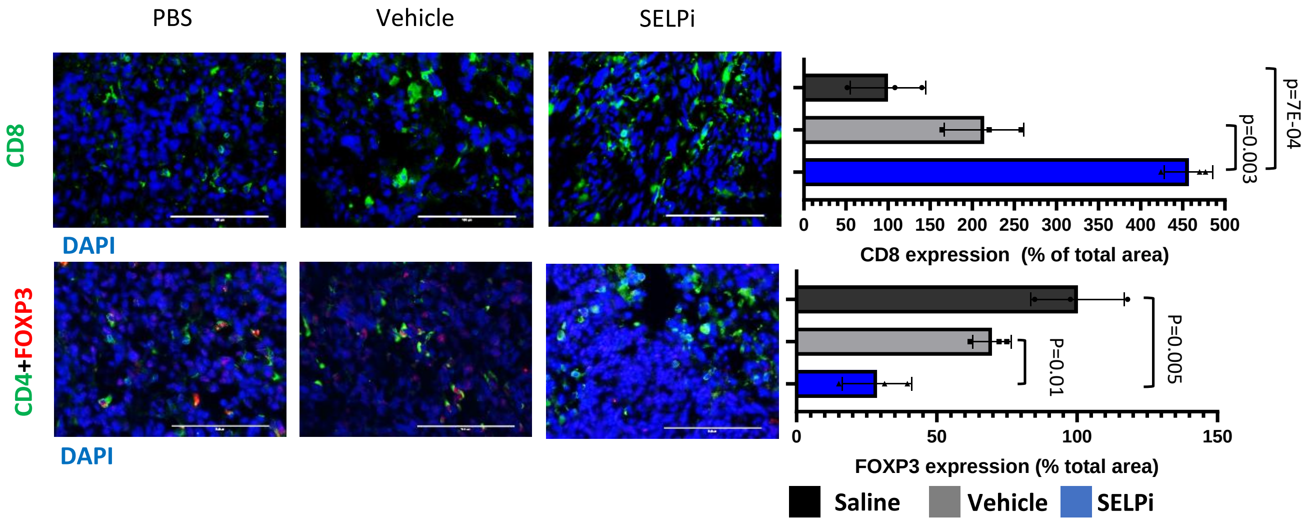

Supplementary Figure 18. SELPi treatment resulted in increased levels cytotoxic T cells and reduced levels of T regs in GL261 tumors. Representative images and quantification of Immunostaining showing high levels of CD8 staining and low levels of FOXP3 staining in SELPi treated tumors compared to saline and vehicle. Data represent mean ± s.d. Each dot represents the average of three images per mouse. N=3 mice per group. Statistical significance was determined using one-way ANOVA test with multiple comparisons adjustment. Scale bars represent 100 µm. Source data are provided as a Source Data file.

Supplementary Figure 19. Systemic treatment with SELPi did not cause significant changes in blood chemistry but affected lymphocytes and neutrophils count

A

Blood chemistry

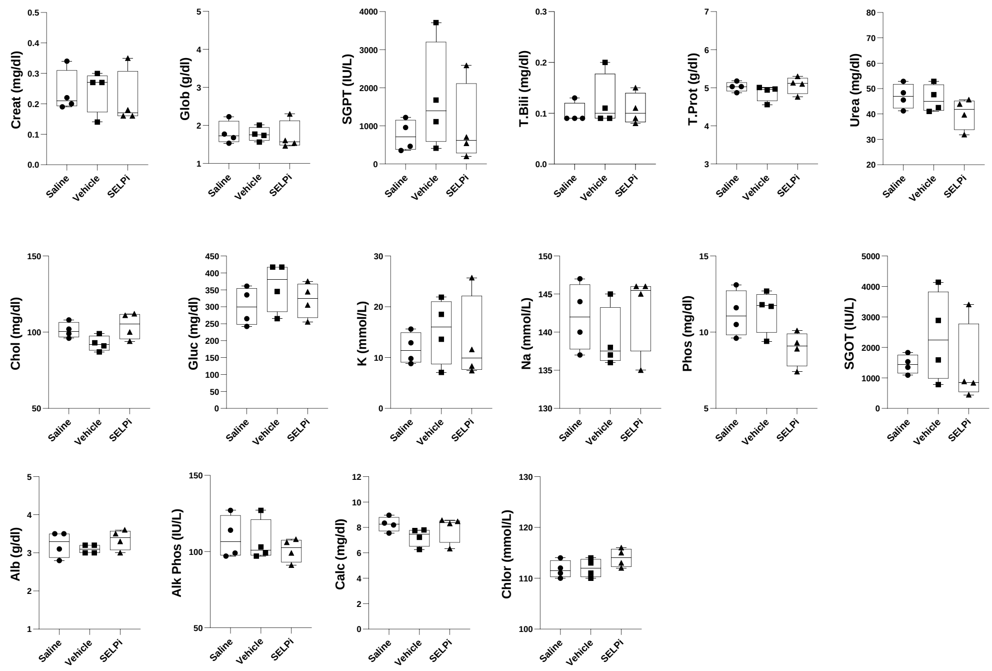

B

Complete blood count

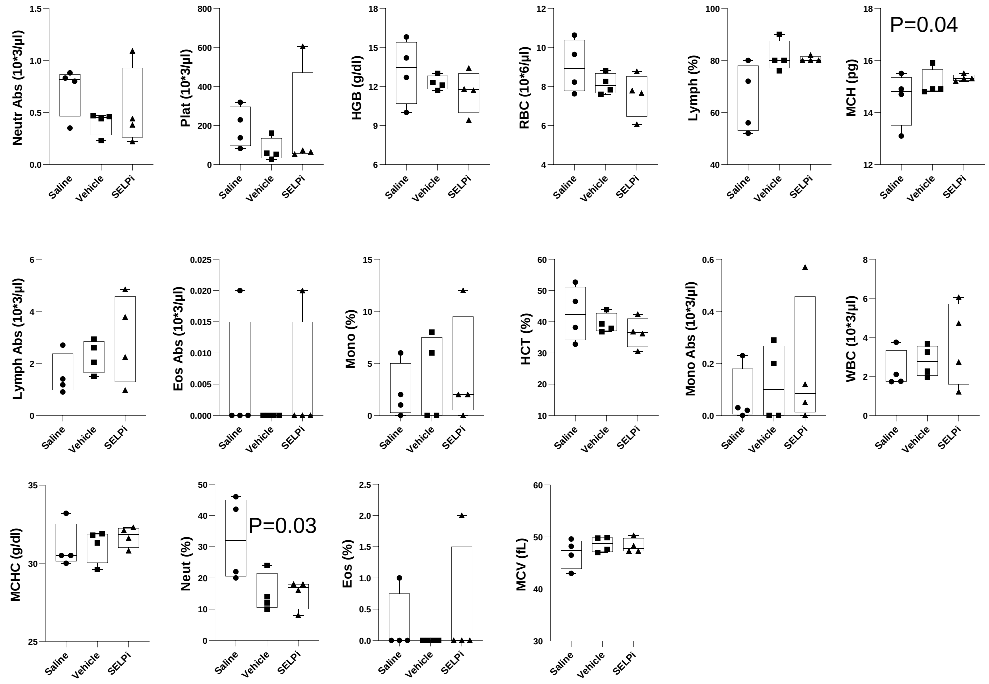

C

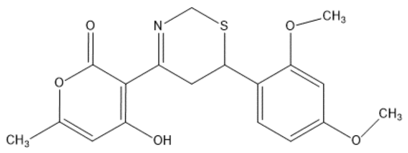

Technical data

**MW**	**375.44**
**Formula**	$C_{19}H_{21}NO_5S$
**Storage**	Desiccate at +4°C
**Purity**	≥97% (HPLC)
**CAS Number**	257292-29-8
**PubChem ID**	5373277
**InChI Key**	FJDFKOSFDPIMBV-NBVRZTHBSA-N
**Smiles**	COC1=CC=C(C2CC(=NCCS2)C2=C(O)C=C(C)OC2=O)C(OC)=C1

Supplementary Figure 19. Systemic treatment with SELPi did not cause significant changes in blood chemistry but affected lymphocytes and neutrophils count. A-B. Naïve mice were treated with a single IV dose of 16 mg/kg SELPi. Following 24 h, blood chemistry (A) and complete blood count analyses (B) were performed, showing no significant changes in any of the evaluated factors compared between the untreated, vehicle- or SELPi- treated groups. Blood count shows elevated levels of lymphocytes (P=0.04) and reduced levels of neutrophils (P = 0.03). N=4 mice per group. P value was calculated by One-way ANOVA test with multiple comparisons adjustment. Center of the box plots shows median values, boxes extent from 25% to the 75% percentile, whiskers show minimum and maximum values. **C.** Molecular scheme drawn by ChemDraw 15.0. Technical data of SELPi as provided by the manufacturer- Tocris (https://www.tocris.com/products/kf-38789_2748).

Supplementary Figure 20. Extracranial tumor growth

A

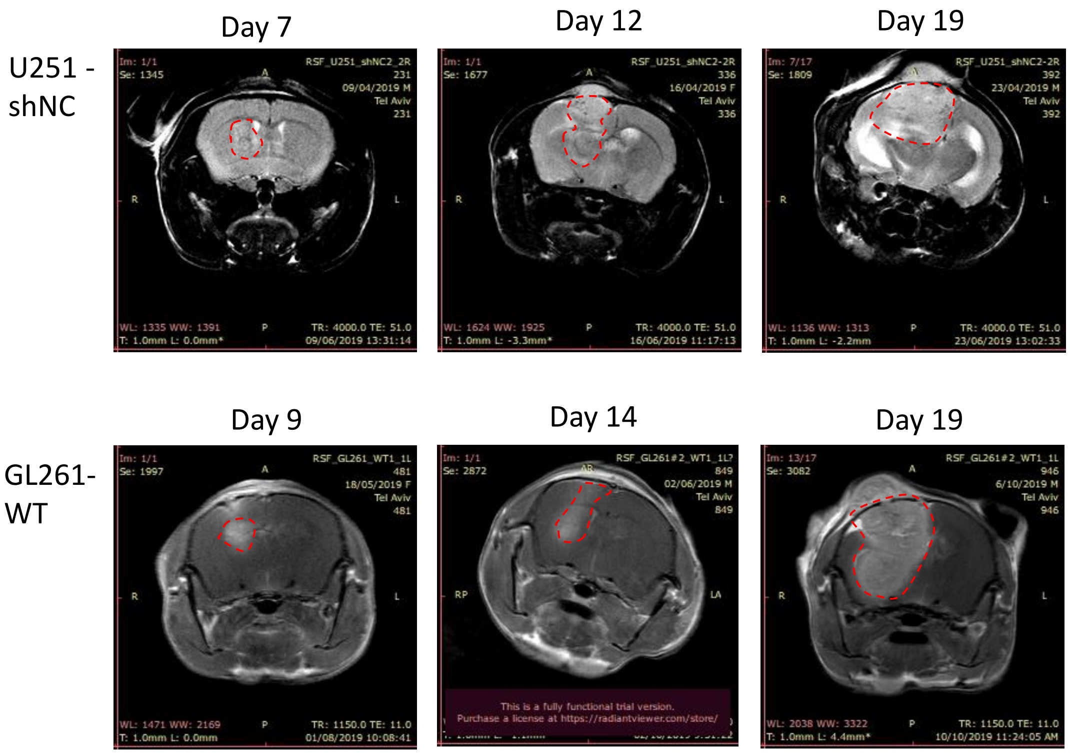

B

	U251 SELP knockdown		
	WT	shNC	shSELP
No	3	3	0
Frequency	1/3	1/2.6	0

	PD-GB4 SELP knockdown		
	WT	shNC	shSELP
No	2	3	0
Frequency	1/4	1/3	0

	GL261 SELP knockdown		
	WT	shNC	shSELP
No	5	7	2
Frequency	1/5.8	1/4.1	1/14.4

	GL261 SELPi treatment		
	WT	shNC	shSELP
No	2	1	1
Frequency	1/2.5	1/5	1/5

	PD-GB4 SELPi treatment		
	WT	shNC	shSELP
No	1	0	0
Frequency	1/5	0	0

	iAGR SELPi treatment		
	WT	shNC	shSELP
No	4	3	1
Frequency	1/3.5	1/3	1/15

Supplementary Figure 20. Extracranial tumor growth. A. Examples of extracranial tumor growth of U251 and GL261 GB tumors which occurred only in late stages of tumor development, following rapid intracranial growth. **B.** A table showing the numbers and frequencies of the cases of extracranial growth in each *in vivo* experiment performed.
