# Supplementary material for: P-selectin axis plays a key role in microglia immunophenotype and glioblastoma progression
Source: Nat Commun. 2021 Mar 26;12:1912. doi: 10.1038/s41467-021-22186-0 (PMC7997963; doi:10.1038/s41467-021-22186-0)
Supplement: Supplementary file 3 — Reporting Summary [file 41467_2021_22186_MOESM3_ESM.pdf]

nature research

Corresponding author(s): Ronit Satchi-Fainaro

Last updated by author(s): 24.2.21

Reporting Summary

Nature Research wishes to improve the reproducibility of the work that we publish. This form provides structure for consistency and transparency in reporting. For further information on Nature Research policies, see Authors & Referees and the Editorial Policy Checklist .

Statistics

For all statistical analyses, confirm that the following items are present in the figure legend, table legend, main text, or Methods section.

n/a	Confirmed	
☐	☒	The exact sample size (*n*) for each experimental group/condition, given as a discrete number and unit of measurement
☐	☒	A statement on whether measurements were taken from distinct samples or whether the same sample was measured repeatedly
☐	☒	The statistical test(s) used AND whether they are one- or two-sided<br>*Only common tests should be described solely by name; describe more complex techniques in the Methods section.*
☐	☒	A description of all covariates tested
☐	☒	A description of any assumptions or corrections, such as tests of normality and adjustment for multiple comparisons
☐	☒	A full description of the statistical parameters including central tendency (e.g. means) or other basic estimates (e.g. regression coefficient) AND variation (e.g. standard deviation) or associated estimates of uncertainty (e.g. confidence intervals)
☐	☒	For null hypothesis testing, the test statistic (e.g. *F*, *t*, *r*) with confidence intervals, effect sizes, degrees of freedom and *P* value noted<br>*Give P values as exact values whenever suitable.*
☒	☐	For Bayesian analysis, information on the choice of priors and Markov chain Monte Carlo settings
☐	☒	For hierarchical and complex designs, identification of the appropriate level for tests and full reporting of outcomes
☒	☐	Estimates of effect sizes (e.g. Cohen's *d*, Pearson's *r*), indicating how they were calculated

Our web collection on statistics for biologists contains articles on many of the points above.

Software and code

Policy information about availability of computer code

Data collection	CellRanger software (v1.3 10X Genomics) and Seurat R package were used for Single cell RNA-seq data collection
Data analysis	For single cell RNA seq analysis- the software used for the analyses of each of the data platforms, and integrated analyses are described and referenced in the individual Method Details subsections. CellRanger software (version 1.3 10X Genomics) and Seurat R version 3.1.4 package were used for QC, analysis, and exploration of the single-cell RNA-seq data.<br>For image analysis- ImageJ 1.52v was used. For MRI tumor volume analysis - Radiant dicom viewer 2020.1.1 was used.<br>For flow cytometry analysis Kaluza analysis 2.1 was used. For drawing chemical structure of SELP inhibitor- ChemDraw Professional version 15.0 was used. The illustration shown in Figure 8 was created with BioRender.com. For graphical presentation and statistical analysis of the results GraphPad prism version 8 was used.

For manuscripts utilizing custom algorithms or software that are central to the research but not yet described in published literature, software must be made available to editors/reviewers. We strongly encourage code deposition in a community repository (e.g. GitHub). See the Nature Research guidelines for submitting code & software for further information.

Data

Policy information about availability of data

All manuscripts must include a data availability statement. This statement should provide the following information, where applicable:

- Accession codes, unique identifiers, or web links for publicly available datasets
- A list of figures that have associated raw data
- A description of any restrictions on data availability

The sequence data generated in this study, assosiated with figure 6 and supplementary figures 13-17, have been deposited in the Gene Expression Omnibus (GEO) and are accessible through the GEO Series accession number GSE156663. The software used for the analyses of each of the data platforms, and integrated analyses are described and referenced in the individual Method Details subsections.

Field-specific reporting

Please select the one below that is the best fit for your research. If you are not sure, read the appropriate sections before making your selection.

- [x] Life sciences
- [ ] Behavioural & social sciences
- [ ] Ecological, evolutionary & environmental sciences

For a reference copy of the document with all sections, see nature.com/documents/nr-reporting-summary-flat.pdf

Life sciences study design

All studies must disclose on these points even when the disclosure is negative.

Sample size	We chose to use sample size of at least three independent repeats which is sufficient to determine statistical significance. For in vitro assays, technical repeats were performed in triplicates for statistical significance of each independent study. Commercial kits as ELISA, Cytokine array and nitric oxide measurements, were performed in duplicates as manufacturer advised, and statistical significance was determined using at least three independent studies. When indicated, statistical significance was tested using at least three technical repeats of a reliable representative experiment of at least three independent studies. For animal studies, all groups contained at least three mice, when possible each group contained at least five mice per group for statistical significance and each in vivo experiment was performed using different models.As indicated in the manuscript, some in vivo experiments included n=10-14 mice per group.<br>In general, the number of animals in each group was determined according to previous studies cited in our manuscript. The size of each sample is in close agreement with those studies already published and with the need for statistical analysis to discuss the degree of differences and measure the variability of these in vivo data.
Data exclusions	In rare cases of technical repeats used for in vitro assays, abnormal values due to technical issues were excluded according to standard deviation of the replicates. There was no exclusion of biological repeat. There was no data exclusion for in vivo assay except in cases when there was no tumor take or technical measurement errors.
Replication	To ensure reproducibility, after experiments calibration, all the experiments were performed in at least three independent studies using the exact same conditions and fresh samples and reagents. All the replication attempts were successful as described in the figures.
Randomization	For in vitro assays, cells were divided from the same initial sample in the same manner using the same conditions. Treatment groups were divided randomly between the plates or wells. Freshly thawed cells were used for repeats. Mice were randomized prior to injections or treatments according to their weight to ensure similar weight average between the groups. Mice chosen for intermediate endpoints were chosen according to tumor size or weight average of each group.
Blinding	As most of the experiments were performed and analyzed by the same person, blinding was mostly not possible. When possible, a table of numbers instead of names was prepared in advanced and data was initially analyzed according to the table. All data collection and analysis software and parameters were identical between groups and all the data of an individual experiment was collected and analyzed at the same time by the person. Blinding was possible in the following experiments: MRI imaging of U251 and PD-GB4 tumors shown in Figure 4A, D. Phagocyte assay shown in Figure 3J, Supplementary Figures 9D and 10E. Cytokine array shown in Supplementary Figure 1C. Flow cytometry analysis shown in Supplementary Figure 12C. Image acquisition of immunostaining of U251 tumors shown in Figure 4F. Real-Time PCR analysis shown in Figure 3F-I and Supplementary Figure 9A-B.

Reporting for specific materials, systems and methods

We require information from authors about some types of materials, experimental systems and methods used in many studies. Here, indicate whether each material, system or method listed is relevant to your study. If you are not sure if a list item applies to your research, read the appropriate section before selecting a response.

Materials & experimental systems

n/a	Involved in the study
[ ]	[x] Antibodies
[ ]	[x] Eukaryotic cell lines
[x]	[ ] Palaeontology
[ ]	[x] Animals and other organisms
[ ]	[x] Human research participants
[x]	[ ] Clinical data

Methods

n/a	Involved in the study
[x]	[ ] ChIP-seq
[ ]	[x] Flow cytometry
[ ]	[x] MRI-based neuroimaging

Antibodies

Antibodies used	Anti-human PSGL-1 neutralizing antibody (Cat. No. MAB3345; Lot. No. CLYK0120111; Clone 688102), and anti-human SELP neutralizing antibody (Cat. No. AF137; Lot. No. FBX0518051) were purchased from R&D Systems (Minneapolis, Minnesota, USA). Human L-507 cytokine array kit (Cat. No. AAH-BLM-1A-4; Lot No. 102920 009) was purchased from RayBiotech (Norcross, Georgia, United States). Anti-human/mouse CD44 neutralizing antibody (Cat. No. NBP2-2530; Lot No. VC289186) was purchased from Novus (Colorado, USA). Anti-murine PSGL-1 neutralizing antibody (Cat. No. BE0188; Lot No. 676818M2) was purchased

from Bio X Cell (Massachusetts, USA). MEBCYTO Apoptosis Kit, was purchased from MBL International (UK). Anti-human (SWA11) and anti-murine (Ml.69) CD24 neutralizing antibodies were kindly provided by Nadir Arber and Shiran (Tel Aviv Sourasky Medical Center). Primary immunostaining antibodies: Rabbit anti-mouse Iba1 (Cat. No. NBP2-19019; Lot. No. 41556; Dilution: 1:200), rat anti-human/mouse PSGL-1 (Cat. No. NB100; Lot. No. C; Dilution 1:50), rabbit anti-human/mouse Ki-67 (Cat. No. NB500-170; Lot. No. G15; Dilution 1:50), and rabbit anti-mouse FOXP3 (Cat. No. NB600; Lot. No. D-1; Dilution 1:30) were purchased from Novus (Colorado, USA). Mouse anti-human SELP (Cat. No. BBA1; Lot. No. APB081704; Clone BBIG-E; Dilution 1:30) was purchased from R&D Systems (Minneapolis, Minnesota, USA). Mouse anti-mouse SELP (Cat. No. 148302; Lot No. B186735; Clone RMP-1; Dilution 1:50) was purchased from BioLegend (San Diego, California, USA). Rat anti-mouse CD31 (Cat. No. 550272; Lot. No. 6273859; Dilution 1:25) was purchased from BD Biosciences (Franklin Lakes, NJ, USA). Rabbit anti-human/mouse Caspase-3 (Cat. No. CST-9664L; Lot. No. 21; Dilution 1:30) was purchased from Cell Signaling Technology (Danvers, Massachusetts, USA). Rat anti-mouse CD4 (Cat. No. 14-9766-82; Lot. No. 4307664; Clone 4SMAS; Dilution 1:100) and Rat anti-CD8 (Cat. No. 14-0808-82; Lot. No. 2003225; Clone: 4SMIS; Dilution 1:50) were purchased from eBioscience (San Diego, California, USA).

Secondary immunostaining antibodies: Goat anti-mouse Alexa Fluor® 647 (Cat. No. ab15115; Lot. No. GR309891-3; Dilution 1:300), goat anti-rabbit Alexa Fluor® 488 (Cat. No. ab150077; Lot No. GR315933-2; Dilution 1:300), and goat anti-rabbit Alexa Fluor® 647 (Cat. No. Ab150079; Lot. No. Gr3176223-2; Dilution 1:300) were purchased from Abcam (Cambridge, United Kingdom). Goat anti-rat Alexa Fluor® 488 (Cat 112-545-068; Lot. No. 143654; Dilution 1:300) and goat anti-rat Alexa Fluor® 647 (Cat. No. 112-605-003; Lot No. 137652; Dilution 1:300) were purchased from Jackson ImmunoResearch Laboratories, Inc. (West Grove, Pennsylvania, USA).

Flow cytometry antibodies: Mouse anti-human SELP (Cat. No. BBA1; Lot. No. APB081704; Clone BBIG-E; Dilution 1:20), Mouse IgG1 isotype control (Cat. No. mab002; Dilution 1:20) were purchased from R&D Systems (Minneapolis, Minnesota, USA). Rat anti-human/mouse PSGL-1 (Cat. No. NB100; Lot. No. C; Dilution 1:24) was purchased from Novus (Colorado, USA). Rabbit anti-human/mouse CD163 (Cat. No. AB182422; Lot. No. GR3232711-5; Dilution 1:20), anti-mouse CD11b (Cat. No. ab8878; Lot. No. GR131048-4; Dilution 1:25), and goat anti-rabbit Alexa Fluor® 488 (Cat. No. ab150077; Lot No. GR315933-2; Dilution 1:50) were purchased from Abcam (Cambridge, United Kingdom). Anti-mouse CD3-FITC (Cat. No. 130-119-798; Lot. No. 5190919162; Clone REA641; Dilution 1:10), anti-mouse CD8-APC (Cat. No. 130-111-712; Lot No. 5190919051; Clone: REA793; Dilution 1:10), anti-mouse CD4 VioBlue® (Cat. No. 130-118-696; Lot No. 5190919087; Clone REA605; Dilution 1:10), REA Control-APC (Cat. No. 130-113-446; Lot. No. 5190711317; Clone REA293; Dilution 1:10), REA Control-VioBlue® (Cat. No. 130-113-545; Lot. No. 5190711335; Clone REA293; Dilution 1:10), anti-mouse CD38-APC-Vio770 (Cat. No. 130-125-227; Lot No. 5200405654; Clone REA616; Dilution 1:50), anti-mouse F4/80-FITC (Cat. No.130-117-509; Lot. Np. 52003066886; Clone REA126; Dilution 1:50), anti-CD11b-PE-Vio770 (Cat No. 130-113-808; Lot No. 5190919070; Clone REA592; Dilution 1:50) and REA Control-FITC (Cat. No. 130-113-449; Lot No. 5190711318; Clone REA293; Dilution 1:10) were purchased from Miltenyi Biotec (Bergisch Gladbach, Germany). Anti-mouse FOXP3 Alexa Fluor® 647 (Cat. No. 126408; Lot. No. B264076; Clone MF-14; Dilution 1:25), Alexa Fluor® 647 IgG2b, k Isotype ctrl (Cat. No. 400626; Lot. No. B243822; Clone RTK4530; Dilution 1:25), anti-mouse P2Y12-PE (Cat. No. 848003; Lot No. B264216; Clone S16007D, Dilution 1:50), anti-human P2Y12-Briliant Violet 421 (Cat No. 392105; Lot No. B286137; Clone S16001E; Dilution 1:50), anti-human TMEM119 (Cat No. 853301; Lot No. B272769; Clone A16075D; Dilution 1:50), anti-mouse CD206-PE (Cat. No. 141706; Lot No. B280038; Clone C068C2; Dilution 1:50) and LEAF Purified Rat IgG2b, k Isotype Ctrl (Cat. No. 400621; Lot. No. B209798; Clone RTK4530) were purchased from BioLegend (San Diego, California, USA). Mouse-IgG k BP-CFL 488 (Cat. No. sc-516176; Lot. No. HO118; Dilution 1:20) was purchased from Santa Cruz Biotechnology, Inc. (Dallas, Texas, USA). Goat anti-rat Alexa Fluor® 488 (Cat 112-545-068; Lot. No. 143654; Dilution 1:50) was purchased from Jackson ImmunoResearch Laboratories, Inc. (West Grove, Pennsylvania, USA). anti-human CD206 (Cat No. 60143-1-ig; Dilution 1:100) and anti-human TMEM119 (Cat. No. 27585-1-ap; Dilution 1:100) were purchased from ProteinTech (Rosemont, California, USA).

Western Blot antibodies: Anti-human total p65 (Cat. No. D14E12; Lot. No. 13; Dilution 1:1000), anti-human phospho p65 (Cat. No. S536; Lot. No. 16; Dilution 1:1000), anti-human vinculine (Cat. No. E1E9V; Lot. No. 6; Dilution 1:1000) and anti-rabbit HRP (Cat. No. 7074P2; Lot. No. 28; Dilution 1:2000) were purchased from Cells Signaling (Massachusetts, USA)

Validation

All antibodies used were validated for the specific application and species by the manufacturers and validation data and references were provided in manufacturers website. All antibodies were used according the manufactures instructions only for the validated species and application. Validation statements on the manufacturer's website for all primary antibodies used: Rabbit anti-mouse/human Iba1 (Cat. No. NBP2-19019): AIF-1/Iba1 antibody validated for ICC/IF from a verified customer review. Rat anti-human/mouse PSGL-1 (Cat. No. NB100): CLA antibody clone HECA-452 is known to recognize the cutaneous lymphocyte-associated antigen / CLA which is a specialized glycosylated form of P-selectin glycoprotein ligand-1/PSGL-1. Rabbit anti-human/mouse Ki-67 (Cat. No. NB500-170): We have publications tested in 4 confirmed species: Human, Mouse, Rat, Porcine. We have publications tested in 6 applications: Flow, ICC/IF, IHC, IHC-Fr, IHC-P, WB. and rabbit anti-mouse FOXP3 (Cat. No. NB600): Mouse thymus shows nuclear staining in some patches of thymocytes. Mouse anti-human SELP (Cat. No. BBA1): Binds to COS cells transfected with human P-Selectin. It does not bind to CHO cells transfected with human ICAM-1, L-Selectin, PECAM-1 or VCAM-1. Mouse anti-mouse SELP (Cat. No. 148302): Each lot of this antibody is quality control tested by immunofluorescent staining with flow cytometric analysis, Additional reported applications (for the relevant formats) include: immunofluorescence, immunohistochemistry, ELISA, Western blotting, immunoprecipitation, and blocking in rats and mice. Rat anti-mouse CD31 (Cat. No. 550272): The MEC13.3 antibody is recommended to test for immunohistochemical staining of Zinc-fixed paraffin sections. Tissues tested were mouse spleen, lung, heart, and thymus. Immunohistochemistry of acetone-fixed frozen sections has been reported. The antibody stains endothelial cells on small and large blood vessels. Rabbit anti-human/mouse Caspase-3 (Cat. No. CST-9664L): Cleaved Caspase-3 (Asp175) (5A1) Rabbit mAb detects endogenous levels of the large fragment (17/19 kDa) of activated caspase-3 resulting from cleavage adjacent to Asp175. This antibody does not recognize full length caspase-3 or other cleaved caspases. Rat anti-mouse CD4 (Cat. No. 14-9766-82): Applications Tested: This 4SM95 antibody has been tested by immunohistochemistry of formalin-fixed paraffin embedded mouse tissue using low or high pH antigen retrieval and can be used at less than or equal to 5 µg/mL. This 4SM95 antibody has also been tested by western blot of reduced mouse spleen lysate and can be used at less than or equal to 5 µg/mL. Rat anti-CD8 (Cat. No. 14-0808-82): Applications Tested: This 4SM15 antibody has been tested by immunohistochemistry of formalin-fixed paraffin embedded mouse tissue using low or high pH antigen retrieval and can be used at less than or equal to 5 µg/mL. The 4SM15 antibody has been tested by flow

cytometric analysis of mouse splenocytes and can be used at less than or equal to 0.25 µg per test. Rabbit anti-human/mouse CD163 (Cat. No. AB182422): Our Abpromise guarantee covers the use of ab182422 in the following tested applications. anti-mouse CD11b (Cat. No. ab8878): Our Abpromise guarantee covers the use of ab8878 in the following tested applications. Anti-mouse CD3-FITC (Cat. No. 130-119-798): Splenocytes from C57BL/6 mice were stained with CD3 antibodies or with the corresponding REA Control antibodies (left images) as well as with CD8a antibodies. Flow cytometry was performed using the MACSQuant® Analyzer. Cell debris and dead cells were excluded from the analysis based on scatter signals and propidium iodide fluorescence or 4',6-diamidino-2-phenylindole (DAPI) fluorescence, as in the case of tandem conjugates. Anti-mouse CD8-APC (Cat. No. 130-111-712): Splenocytes from BALB/c mice were stained with CD8b antibodies or with the corresponding REA Control antibodies (left image) as well as with CD3ε antibodies. Flow cytometry was performed using the MACSQuant® Analyzer. Cell debris and dead cells were excluded from the analysis based on scatter signals and propidium iodide fluorescence or 4',6-diamidino-2-phenylindole (DAPI) fluorescence, as in the case of tandem conjugates. Anti-mouse CD4 VioBlue® (Cat. No. 130-118-696): splenocytes of BALB/c mice were stained with CD4 antibodies or with the corresponding REA Control antibodies (left images) as well as with CD3ε antibodies. Flow cytometry was performed using the MACSQuant® Analyzer. Anti-mouse CD38-APC-Vio770 (Cat. No. 130-125-227): Splenocytes from BALB/c mice were stained with CD38 antibodies or with the corresponding REA Control antibodies (left peak). Flow cytometry was performed using the MACSQuant® Analyzer. Anti-mouse F4/80-FITC (Cat. No.130-117-509): Spleen cells from BALB/c mouse were stained with Anti-F4/80 antibodies as well as with Anti-Ly-6C antibodies and analyzed by flow cytometry using the MACSQuant® Analyzer. CD45.2+ cells were pre-gated for the analysis. anti-CD11b-PE-Vio770 (Cat No. 130-113-808): Splenocytes from BALB/c mice were stained with CD11b antibodies or with the corresponding REA Control antibodies (left images). Flow cytometry was performed using the MACSQuant® Analyzer. Anti-mouse FOXP3 Alexa Fluor® 647 (Cat. No. 126408): Each lot of this antibody is quality control tested by intracellular flow cytometry using our True-Nuclear™ Transcription Factor Staining Protocol. Anti-mouse P2Y12-PE (Cat. No. 848003): Each lot of this antibody is quality control tested by immunofluorescent staining with flow cytometric analysis. Anti-human TMEM119 (Cat No. 853301): Each lot of this antibody is quality control tested by immunofluorescent staining with flow cytometric analysis. Anti-mouse CD206-PE (Cat. No. 141706): Each lot of this antibody is quality control tested by intracellular immunofluorescent staining with flow cytometric analysis. Anti-human CD206 (Cat No. 60143-1-ig): Tested Applications: Positive WB detected in human placenta tissue, human liver tissue. Positive IP detected in human placenta tissue. Positive IHC detected in human lung cancer tissue, human liver tissue. Positive FC detected in RAW 264.7 cells.

Eukaryotic cell lines

Policy information about cell lines

Cell line source(s)	U251 human GB cell line was obtained from the European Collection of Authenticated Cell Cultures (ECACC) (Porton Down, Salisbury, UK). GL261 murine GB cell line was obtained from the National Cancer Institute (Frederick, MD, USA). Primary human microglia were obtained from Celprogen (Torrance, California, USA). Human embryonic kidney 293T cells (HEK 293T) were obtained from the American Type Culture Collection (ATCC, Manassas, VA, USA). iAGR53, PNp53 and EGFRviii-shP16 murine GB cell lines were generated by lentiviral vector as previously described (Marumoto, T., et al., Development of a novel mouse glioma model using lentiviral vectors. Nature Medicine, 2009. 15(1): p. 110-116.<br>40. Friedmann-Morvinski, D., et al., Dedifferentiation of neurons and astrocytes by oncogenes can induce gliomas in mice. Science (New York, N.Y.), 2012. 338(6110): p. 1080-1084).
Authentication	U251 cell line was authenticated by the European Collection of Authenticated Cell Cultures (ECACC) using morphology, karyotyping, PCR-based techniques, and Cytocrome oxidase I assay, following manufacturer validated procedures. iAGR53, PNp53 and EGFRviii-shP16 murine GB cell lines were not authenticated by the authors.
Mycoplasma contamination	Cells were routinely tested for mycoplasma contamination with a mycoplasma detection kit (Biological Industries, Israel). All cell lines used were tested negative for mycoplasma contamination.
Commonly misidentified lines<br>(See ICLAC register)	We did not use any misidentified lines.

Animals and other organisms

Policy information about studies involving animals; ARRIVE guidelines recommended for reporting animal research

Laboratory animals	Six week-old male C57BL/6 mice and six week-old male SCID mice were used. Groups of up to five mice per IVC cage (Lab-Products) were housed on a 12 h light/dark cycle, on autoclaved ASPEN wood chips bedding, at an ambient temperature of 22°C ±1°C, with humidity controlled at 50%, had ad libitum access to regular laboratory chow (Altromin1324; Altromin, Lage, Germany), and were provided with UV-irradiated and micro-filtered Hydropac system for water.
Wild animals	The study did not involve wild animals
Field-collected samples	The study did not involve field-collected samples
Ethics oversight	Animals were housed in the Tel Aviv University animal facility. All experiments were approved by the animal care and use committee (IACUC) of Tel Aviv University (approval no. 01-19-015, 01-19-097) and conducted in accordance with NIH guidelines.

Note that full information on the approval of the study protocol must also be provided in the manuscript.

Human research participants

Policy information about studies involving human research participants

Population characteristics	FFPE GB samples were obtained from Tel Aviv Sourasky Medical Center. A total of 60 samples were collected: 36 samples of patients who survived short-term- STS (69% men; 65 ± 2 years; survival of 3.7 ± 0.2 months), and 24 samples of patients who survived long-term- LTS (58% men; 56 ± 3 years; survival of 48 ± 3.9 months). IRB approval no. 0735-13-TLV. Healthy human brain samples were collected by Thomas Hyde at the Lieber Institute as described in the manuscript under an approved IRB protocol No. 90-M-0142.
Recruitment	FFPE samples from GB patients were chosen according to patient's survival rates (covering large spectrum from short-term to long-term male and female survivors). For healthy human samples, Clinical characterization, diagnoses, and macro- and microscopic anthropological examinations were performed on all samples using a standardized paradigm, and subjects with evidence of macro- or microscopic neuropathology were excluded.
Ethics oversight	GB patient tissues: experiments involving human GB tissues were performed following an informed consent, with the approval of the Institutional Review Board (IRB) and in compliance with all legal and ethical considerations for human subject research (approval no. 0735-13-TLV). Healthy human brain tissues: Post-mortem human brain tissue was obtained by autopsy from the Offices of the Chief Medical Examiner of the District of Columbia, and of the Commonwealth of Virginia, Northern District, all with informed consent from the legal next of kin (protocol 90-M-0142 approved by the NIMH/NIH IRB). Additional post-mortem human brain tissue samples were provided by the National Institute of Child Health and Human Development Brain and Tissue Bank for Developmental Disorders (http://www.BTBank.org) under contracts NO1-HD-4-3368 and NO1-HD-4-3383. The IRB of the University of Maryland at Baltimore and the State of Maryland approved the protocol, and the tissue was donated to the Lieber Institute for Brain Development under the terms of a Material Transfer Agreement. Clinical characterization, diagnoses, and macro- and microscopic neuropathological examinations were performed on all samples using a standardized paradigm, and subjects with evidence of macro- or microscopic neuropathology were excluded. Details of tissue acquisition, handling, processing, dissection, clinical characterization, diagnoses, neuropathological examinations, RNA extraction, and quality control measures were as described previously [56]. The Brain and Tissue Bank cases were handled in a similar fashion (http://medschool.umaryland.edu/BTBank/ProtocolMethods.html).

Note that full information on the approval of the study protocol must also be provided in the manuscript.

Flow Cytometry

Plots

Confirm that:

- [x] The axis labels state the marker and fluorochrome used (e.g. CD4-FITC).
- [x] The axis scales are clearly visible. Include numbers along axes only for bottom left plot of group (a 'group' is an analysis of identical markers).
- [x] All plots are contour plots with outliers or pseudocolor plots.
- [x] A numerical value for number of cells or percentage (with statistics) is provided.

Methodology

Sample preparation	Cells were harvested using a cell scraper, and were then washed with PBS followed by additional washes with PBS supplemented with 1% BSA and 5 mM EDTA (FACS buffer). Tumor spheroids were recovered from Matrigel using Cell Recovery Solution (Corning) and washed with FACS buffer. Cells were incubated with fluorescence labeled primary antibody for 1 h on ice or incubated with unlabeled primary antibody for 1 h on ice, washed with FACS buffer and incubated with fluorescence labeled secondary antibody for 1 h on ice. Cells were then washed again with FACS buffer and flow cytometry analysis was performed.
Instrument	Fluorescence intensity was assessed using either an Attune flow cytometer (Life Technologies) or a GalliosTM flow cytometer (Beckman Coulter, USA)
Software	Kaluza software (Beckman Coulter, USA) was used for all flow cytomentry experiments.
Cell population abundance	No sorting was performed
Gating strategy	For Gating, density plot of FSC on SSC was generated and most of the population was gated with the exclusion of dead cells and debris. Then, a plot of FSC-A on FSC-H was generated for doublet discrimination, linear gate was performed for singlets gating. Negative and positive gates were performed according to the isotype control group which was defined as 100% negative.

- [x] Tick this box to confirm that a figure exemplifying the gating strategy is provided in the Supplementary Information.

Magnetic resonance imaging

Experimental design

Design type	Mice were imaged at resting state
Design specifications	Each mouse was anesthetized and imaged once using T1 weighted sequence after Gadolinium injection or T2 weighted sequence with no contrast agent. Scans were approximately five minutes long.
Behavioral performance measures	No Behavioral performance measures were performed. Mice were anesthetized and respiratory functions were monitored during the scan.

Acquisition

Imaging type(s)	Structural T1 or T2 weighted images were obtained.
Field strength	4.7 Tesla
Sequence & imaging parameters	T1 weighted, average-4, RF pules-1, TR-1150, Echo spacing-11, TE-11, Echo train-4, PE order-1, total slices-17, thickness-1mm, field of view-25, orientation- coronal. T2 weighted, average-2, RF pules-1, TR-4000, Echo spacing-17, TE-51, Echo train-7, PE order-0, total slices-17, thickness-1mm, field of view-25, orientation- coronal.
Area of acquisition	Whole brain scans were performed. Area was defined by a "scout" scanning prior to T1 or T2 sequences.

Diffusion MRI ☐ Used ☒ Not used

Preprocessing

Preprocessing software	Radiant software was used to view and analyze images. Positive tumor areas were measured and calculated using measurements tools for each slice and a calculation of total tumor volume per mouse was performed.
Normalization	Data was not normalized as there is no need in normalization for simple tumor volume evaluation.
Normalization template	The data was not normalized
Noise and artifact removal	Respiratory gating was performed.
Volume censoring	Since fMRI or other advanced techniques were not performed in this study, and only T1 or T2 weighted scans were performed in order to determine tumor volume, volume censoring is not applicable for this study. Since this is used to reduce motion artifacts, it is not relevant in this case as the mice were anesthesized for these T1 and T2 weighted.

Statistical modeling & inference

Model type and settings	Since only T1 or T2 weighted scans were performed in order to determine tumor volume, this is not applicable for this study. No modelling nor fMRI were performed in this case.
Effect(s) tested	The effect of different treatments on tumor growth was tested. ANOVA test was used to determine statistical significant.

Specify type of analysis: ☒ Whole brain ☐ ROI-based ☐ Both

Statistic type for inference (See Eklund et al. 2016)	Since only T1 or T2 weighted scans was performed in order to determine tumor volume, this is not applicable for this study.
Correction	Since only T1 or T2 weighted scans was performed in order to determine tumor volume, this is not applicable for this study.

Models & analysis

n/a	Involved in the study
☒	☐ Functional and/or effective connectivity
☒	☐ Graph analysis
☒	☐ Multivariate modeling or predictive analysis
